# Giant non-linear susceptibility of hydrogenic donors in silicon and germanium

**DOI:** 10.1038/s41377-019-0174-6

**Published:** 2019-07-10

**Authors:** Nguyen H. Le, Grigory V. Lanskii, Gabriel Aeppli, Benedict N. Murdin

**Affiliations:** 1 Advanced Technology Institute and SEPNet, University of Surrey, Guildford, GU2 7XH UK; 2 Institute of Monitoring of Climatic and Ecological Systems SB RAS, 10/3 Academical Ave., Tomsk, 634055 Russia; 3 Laboratory for Solid State Physics, ETH Zurich, Zurich, CH-8093 Switzerland; 4 Institut de Physique, EPF Lausanne, Lausanne, CH-1015 Switzerland; 5 Paul Scherrer Institut, Villigen, PSI CH-5232 Switzerland

**Keywords:** Terahertz optics, Nonlinear optics, Silicon photonics

## Abstract

Implicit summation is a technique for the conversion of sums over intermediate states in multiphoton absorption and the high-order susceptibility in hydrogen into simple integrals. Here, we derive the equivalent technique for hydrogenic impurities in multi-valley semiconductors. While the absorption has useful applications, it is primarily a loss process; conversely, the non-linear susceptibility is a crucial parameter for active photonic devices. For Si:P, we predict the hyperpolarizability ranges from *χ*^(3)^/*n*_3D_ = 2.9 to 580 × 10^−38^ m^5^/V^2^ depending on the frequency, even while avoiding resonance. Using samples of a reasonable density, *n*_3D_, and thickness, *L*, to produce third-harmonic generation at 9 THz, a frequency that is difficult to produce with existing solid-state sources, we predict that *χ*^(3)^ should exceed that of bulk InSb and *χ*^(3)^*L* should exceed that of graphene and resonantly enhanced quantum wells.

## Semiconductor physics: Giant nonlinearity

Lightly doped semiconductors may provide a mean for highly efficient third-harmonic generation (THG) of terahertz radiation. That’s the hopeful prediction of some theoretical analysis performed by scientists in the UK, Switzerland and Russia. Nguyen Le and coworkers calculated that silicon and germanium featuring hydrogenic impurities should possess a giant nonlinear susceptibility in the terahertz region. The finding potentially opens the door to efficient nonlinear processes, such as THG, in the terahertz region. In principle this means that these semiconductors could be used to frequency triple the emission from 2 to 4 THz quantum cascade lasers, resulting in sources in the 6–12 THz gap. At present, no compact, low-cost sources currently exist in this region and researchers are forced to rely on free-electron lasers and difference frequency generators instead.

## Introduction

Multiphoton absorption requires a high intensity, and was first observed shortly after the invention of the laser using impurities in solids^1^ and alkali vapor^2^. Although multiphoton absorption is useful for metrology and modulators, and can be enhanced where there is near-resonance of an intermediate state as in the case of Rb^3^, it is essentially a loss process contributing an imaginary part to the non-linear susceptibility. The corresponding real part is responsible for a great variety of wavelength conversion processes such as harmonic generation, first observed in quartz^4^ and later in atomic vapors^5^ including alkalies^6^. THz multiphoton absorption has been shown to be very large in hydrogenic shallow impurities in semiconductors, even without intermediate state resonances^7^, due to the large dielectric screening and low effective mass. Here, we predict giant values for the real part of the THz non-linear susceptibility for doped silicon and germanium. This finding opens access to novel applications for these materials in THz photonics. For example, tripling the output of a 2–4 THz quantum cascade laser through third-harmonic generation would fill the frequency gap currently only filled by larger, more expensive systems. We show that a good efficiency can be obtained for third-harmonic generation with doped silicon and germanium. Our theory can be readily applied to any donor in any semiconductor host where the effective mass approximation is valid, and our discussion makes it clear that a giant value of *χ*^(3)^ is expected for donors with a small binding energy in a host with a large dielectric constant and small effective mass.

The theory developed in this paper is appropriate for frequencies both near to and far from loss-inducing resonances, including the effects of effective mass anisotropy, multi-valley interactions and the central cell correction. The method could easily be applied to other systems with complicated potentials, such as multi-quantum wells. Although this work focuses on perturbative harmonic generation, we anticipate that shallow impurities may also be useful for non-perturbative high-harmonic generation (HHG)^8,9^ taking advantage of the excellent control over the carrier-envelope phase of few-cycle pulses in this THz regime, which can be used to enhance HHG^10^.

## Results

### The implicit summation technique

From *N*th-order perturbation theory^7,11^ the N-photon absorption (NPA) transition rate may be written as

1 $$w^{(N)} = 2\pi \frac{{\left( {2\pi \alpha _{fs}} \right)^N}}{N}\left| {M^{(N)}} \right|^2\left[ {\frac{{E_H^2}}{{\varepsilon _r^{N/2}I_a^N}}} \right]\frac{{I_m^N{\mathrm{\Gamma }}^{(N)}}}{{\hbar ^2}}$$

where $I_a = E_H^2/\hbar a_B^2$, *a*_*B*_ is the Bohr radius, *E*_*H*_ the Hartree energy, and *α*_*fs*_ the fine structure constant. *M*^(*N*)^ is a dimensionless transition matrix element, and *I*_*m*_ is the intensity of the light in the medium with relative dielectric permittivity *ε*_*r*_. The lineshape function Γ^(*N)*^(*ω*) has unit area. For silicon and germanium donors, the factors inside the bracket are renormalized, and of particular importance here *I*_*a*_ is ten orders of magnitude smaller for silicon than it is for hydrogen. This is apparent from the formulae of the Hartree energy and Bohr radius for donors in these materials: $E_H = m_t(e^2/4\pi \epsilon _0\varepsilon_r \hbar )^2$, and $a_B = 4\pi \epsilon _0\varepsilon_r \hbar ^2/m_te^2$, where *m*_*t*_ is the transverse effective mass and $\varepsilon_r$ the dielectric constant^12^. Both germanium and silicon have a s*m*all *m*_*t*_ and large $\varepsilon_r$, raising the Bohr radius and lowering the binding energy. The wavefunction is therefore significantly larger than that of alkali atoms, leading to an enhanced dipole matrix element and hence a substantially stronger interaction with light.

The details of the spectrum given by Eq. (1) are controlled by *M*^(*N*)^, which is influenced in silicon by the indirect valley structure, the anisotropic effective mass, and the donor central cell correction potential. Our main aim here is to calculate these effects. For single-photon absorption (*N* = 1) between states |*ψ*_*g*_〉 (the ground state) and |*ψ*_*e*_〉 (the excited state), $M^{(1)} = \left\langle {\psi _e\left| {{\boldsymbol{\epsilon }}.{\boldsymbol{r}}} \right|\psi _g} \right\rangle /a_B$, where ${\bf{\epsilon }}$ is a unit vector in the polarization direction, and Eq. (1) reduces to Fermi’s golden rule. For two-photon absorption,

$$M^{(2)} = \frac{{E_H}}{{\hbar a_B^2}}\mathop {\sum}\limits_j {\frac{{\left\langle {\psi _e\left| {{\boldsymbol{\epsilon }}.{\boldsymbol{r}}} \right|j} \right\rangle \left\langle {j\left| {{\boldsymbol{\epsilon }}.{\boldsymbol{r}}} \right|\psi _g} \right\rangle }}{{\omega _{jg} - \omega _{eg}/2}}}$$

in the ***E***.***r*** gauge, which may be written as *M*^(2)^ = 〈*ψ*_*e*_|*ζG*_1_*ζ*|*ψ*_*g*_〉 where $\zeta = {\boldsymbol{\epsilon }}.{\boldsymbol{r}}/a_B$,

2 $$G_n = \frac{{E_H}}{\hbar }\mathop {\sum }\limits_j \frac{{\left| j \right\rangle \left\langle j \right|}}{{\left( {\omega _{jg} - n\omega } \right)}}$$

and *ω* = *ω*_*eg*_/*N*. The states |*j*〉 are intermediate states, and along with |*ψ*_*e*_〉 & |*ψ*_*g*_〉 they are eigenstates of $H\left| j \right\rangle = \hbar \omega _j\left| j \right\rangle$, where *H* is the Hamiltonian in the dark. For general multiphoton absorption,

3 $$M^{(N \ge 2)} = \left\langle {\psi _e\left| {\zeta G_{N - 1}\zeta \ldots \zeta G_2\zeta G_1\zeta } \right|\psi _g} \right\rangle$$

The summation in Eq. (2) can be avoided^11^ by noticing that (*H* − *W*_*n*_)*G*_*n*_ = *E*_*H*_, where *W*_*n*_ = ℏ*ω*_*g*_ + *n*ℏ*ω*, and *ω* = *ω*_*eg*_/*N* as already mentioned, and by using the completeness relation $\mathop {\sum}\nolimits_j {\left| j \right\rangle \left\langle j \right|} = 1$. In other words,

4 $$G_n = E_H\left( {H - W_n} \right)^{ - 1}$$

Rewriting Eq. (3), *M*^(*N*)^ = 〈*ψ*_*e*_|*ζ*|*ψ*_*N*−1_〉 where |*ψ*_0_〉 = |*ψ*_*g*_〉 and |*ψ*_*n*_〉 is the solution of the partial differential equation (PDE) $G_n^{ - 1}\left| {\psi _n} \right\rangle = \zeta \left| {\psi _{n - 1}} \right\rangle$. Instead of finding *M*^(*N*)^ by repeated application of Eq. (2), which requires infinite sums (that might be reduced down to a few terms if there are obvious resonances), we may now use Eq. (4) and the PDE at each stage, which can be simpler.

The Nth-order susceptibility far from any multiphoton resonances may also be calculated using the Nth-order perturbation theory^13^. For example, the “resonant” term in the third-order susceptibility, *χ*^(3)^(3*ω*), is

$$\frac{{n_{\text{3D}}e^4}}{{\epsilon _0\hbar ^3}}\mathop {\sum }\limits_{l,k,j} \frac{{\left\langle {\psi _g\left| {{\boldsymbol{\epsilon }}.{\boldsymbol{r}}} \right|l} \right\rangle \left\langle {l\left| {{\boldsymbol{\epsilon }}.{\boldsymbol{r}}} \right|k} \right\rangle \left\langle {k\left| {{\boldsymbol{\epsilon }}.{\boldsymbol{r}}} \right|j} \right\rangle \left\langle {j\left| {{\boldsymbol{\epsilon }}.{\boldsymbol{r}}} \right|\psi _g} \right\rangle }}{{(\omega _{lg } - 3\omega )(\omega _{kg} - 2\omega )(\omega _{jg} - \omega )}}$$

where *e* is the electron charge, and *n*_3D_ is the concentration. *χ*^(3)^ may be written in a similar form to Eqs (1) and (3), and for *N*^th^ order,

5 $$\chi ^{(N)} = C^{(N)}\left[ {\frac{{a_B}}{{I_a^{N/2}}}} \right]\frac{{n_{\text{3D}}e^{N + 1}}}{{\hbar ^{N/2}\epsilon _0}}$$

where *C*^(*N*)^ = 〈*ψ*_*g*_|*ζG*_*N*_…*G*_2_*ζG*_1_*ζ*|*ψ*_*g*_〉 is a dimensionless matrix element that may be found in a similar way to *M*^(*N*)^, either by repeated application of Eq. (2)—as has been done previously for alkali metal vapors^6^—or by using the implicit summation method of Eq. (4) with the only difference being *ω* ≠ *ω*_*eg*_/*N*. The antiresonant terms^13^ and other non-linear processes, such as sum-frequency generation, can be calculated with simple modifications to *W*_*n*_ at each step.

### Multi-valley theory for donors in silicon and germanium

In this section, we develop the multi-valley theory for the nonlinear optical processes of donors based on the effective mass approximation (EMA). For simplicity of presentation, we describe the derivation for silicon; the case of germanium is discussed in the Supplementary Materials. It will become apparent that our theory is readily applicable to any donor in any host as long as the EMA is reliable.

To apply the method to donors, we require |*ψ*_*g*_〉, *ω*_*g*_, |*ψ*_*e*_〉, *ω*_*e*_ and *H*|*ψ*_*n*_〉. Silicon and germanium are indirect with equivalent conduction band minima (valleys) near the Brillouin zone edge; each minimum is characterized by a Fermi surface that is a prolate ellipsoid with transverse & longitudinal effective masses, *m*_*t*,*l*_. According to the Kohn-Luttinger effective mass approximation^14^, the state |*ψ*_*j*_〉 of a shallow donor can be decomposed into slowly varying hydrogenic envelope functions, one for each valley, modulated by plane-wave functions corresponding to the crystal momenta at the minima, ***k***_*μ*_ (and a lattice periodic function that is unimportant here). We write $\psi _j({\boldsymbol{r}}) = \mathop {\sum}\nolimits_\mu {e^{i{\boldsymbol{k}}_\mu .{\boldsymbol{r}}}F_{j,\mu }({\boldsymbol{r}})}$ where *F*_*j*,*μ*_(***r***) is the slowly varying envelope function. We have neglected the lattice periodic part, *u*_*μ*_(***r***), of the Bloch functions for the simplicity of presentation. A rigorous derivation with *u*_*μ*_(***r***) included is provided in the Supplementary Materials, but it does not lead to any change in the final equations for the envelope functions (Eqs (7) and (8) below).

We separate the potential into the slowly varying Coulomb term of the donor *V*(***r***), and a rapidly varying term due to the quantum defect that is short range, *U*(***r***), referred to as the central cell correction (CCC). Within the EMA, the kinetic energy term in the Hamiltonian operates only on the envelope function, and the EMA Schrodinger equation may be written as

6 $$\mathop {\sum}\limits_\mu {e^{i{\boldsymbol{k}}_\mu .{\boldsymbol{r}}}} \left[ {H_0 + U - \hbar \omega _j} \right]F_{j,\mu }(r) = 0$$

where *H*_0_ includes the Coulomb potential *V*(***r***): $E_H^{ - 1}H_0 = - \frac{1}{2}a_B^2\left[ {\partial _x^2 + \partial _y^2 + \gamma \partial _z^2} \right] - a_Br^{ - 1}$ using a valley-specific coordinate system (*x*, *y*, *z* where *z* is the valley axis, i.e., the valley-frame is rotated relative to the lab-frame of *x*_1_, *x*_2_, *x*_3_). The kinetic energy has cylindrical symmetry because *γ* = *m*_*t*_/*m*_*l*_ ≠ 1, and *V*(***r***) and *U*(***r***) are spherical and tetrahedral respectively. *H*_0_ produces wave functions that are approximately hydrogen-like, and *U*(***r***) mixes them to produce states that transform as the A_1_, E and T_2_ components of the T_*d*_ point group.

We take *U*(***r***) to be very short range, and we neglect the small change in the envelope functions over the short length scale 2*π*/|***k***_*μ*_|. Premultiplying Eq. (6) by $e^{ - i{\boldsymbol{k}}_{\mu \prime }.{\boldsymbol{r}}}$ and averaging over a volume (2*π*/|***k***_*μ*_|)^3^ around ***r***, the Schrodinger eqn now reads $\left[ {H_0 - \hbar \omega _j} \right]F_{j,\mu }({\boldsymbol{r}}) + \mathop {\sum}\nolimits_{\mu \prime } {U_{\mu \mu \prime }\delta ({\boldsymbol{r}})F_{j,\mu \prime }({\boldsymbol{r}})} = 0$, where *δ*(***r***) is the Dirac delta function, and $U_{\mu \mu \prime } = {\int} {d{\boldsymbol{r}}{\kern 1pt} e^{i({\boldsymbol{k}}_{\mu \prime } - {\boldsymbol{k}}_\mu ).{\boldsymbol{r}}}U({\boldsymbol{r}})}$. For an A_1_ state, all the envelope functions have the same amplitude at *r* = 0, hence, $\mathop {\sum}\nolimits_{\mu \prime } {U_{\mu \mu \prime }\delta ({\boldsymbol{r}})F_{j,\mu \prime }({\boldsymbol{r}})} = - U_{cc}\delta ({\boldsymbol{r}})F_{j,\mu }({\boldsymbol{r}})$, where $U_{cc} = - \mathop {\sum}\nolimits_{\mu \prime } {U_{\mu \mu \prime }}$. It is found experimentally that for E and T_2_ states, the CCC has a rather small effect, and so we neglect it. Since *H*_0_ has cylindrical symmetry, the component of angular momentum about the valley axis is a conserved quantity, i.e., *F*_*j*,*μ*_(***r***) = *e*^*imϕ*^*f*_*j*,*m*,*μ*_(*r*, *θ*), where *m* is a good quantum number, and now *f*_*j*,*m*,*μ*_ is a 2D function only. Substituting into the Schrodinger eqn, premultiplying by *e*^−*im*′*ϕ*^ and finally integrating over *ϕ*, the eigenproblems are

7 $$\begin{array}{*{20}{l}} {\left[ {H_0^{(m)} - U_{cc}\delta (r) - \hbar \omega _j} \right]f_{j,m,\mu }^{(A_1)}(r,\theta )} \hfill & = \hfill & {0} \hfill \\ {\left[ {H_0^{(m)} - \hbar \omega _j} \right]f_{j,m,\mu }^{(E,T_2)}(r,\theta )} \hfill & = \hfill & {0} \hfill \end{array}$$

where $H_0^{(m)} = H_0 + E_Ha_B^2m^2$/2(*r* sin *θ*)^2^. We solve Eq. (7) using a 2D finite element method (FEM) (see Supplementary Materials).

We focus on silicon, in which case the valley index, *μ*, runs over (±1, ±2, ±3), where 1, 2, 3 are the three crystal axes, and we let the light be polarized along a crystal axis, *x*_1_, by way of illustration; the calculation for germanium and other polarization directions is described in the Supplementary Materials. For the *μ* = ±1, ±2, ±3 valleys, *a*_*B*_*ζ*_*μ*_ = *z*, *x*, *y* = *r* cos *θ*, *r* sin *θ* cos *ϕ*, *r* sin *θ* sin *ϕ*, respectively, because each has its coordinate rotated so that *z* is the valley axis. Following the expansion of *ψ*_*j*_ in terms of the *f*_*j*,*m*,*μ*_, we write the intermediate state functions as $\psi _n({\boldsymbol{r}}) = \mathop {\sum}\nolimits_{m,\mu } {e^{im\phi }e^{i{\boldsymbol{k}}_\mu .{\boldsymbol{r}}}f_{n,m,\mu }(r,\theta )}$, substitute them into $G_n^{ - 1}\psi _n = \zeta \psi _{n - 1}$, premultiply by $e^{ - i{\boldsymbol{k}}_{\mu \prime }.{\boldsymbol{r}}}$, average over a volume of (2*π*/|**k**_*μ*_|)^3^, premultiply by *e*^−*im*′*ϕ*^, and finally, integrate over *ϕ*. Since *f*_0,0,*μ*_ = *f*_*g*,0,*μ*_ for all *μ*, we find that *f*_*n*,*m*,3_ = *i*^−*m*^*f*_*n*,*m*,2_ and *f*_*n*,*m*,−*μ*_ = *f*_*n*,*m*,*μ*_, and

8 $$\begin{array}{*{20}{l}} {\left[ {H_0^{(m)} - W_n - {\cal{D}}} \right]f_{n,m,1} - 2{\cal{D}}f_{n,m,2} = (E_H/a_B){\kern 1pt} r{\kern 1pt} {\mathrm{cos}}{\kern 1pt} \theta f_{n - 1,m,1}} \hfill \\ {\left[ {H_0^{(m)} - W_n - 2{\cal{D}}} \right]f_{n,m,2} - {\cal{D}}f_{n,m,1} = (E_H/a_B){\kern 1pt} r{\kern 1pt} {\mathrm{sin}}{\kern 1pt} \theta \left[ {f_{n - 1,m - 1,2} + f_{n - 1,m + 1,2}} \right]/2} \hfill \end{array}$$

where ${\cal{D}} = U_{cc}\delta ({\boldsymbol{r}})\delta _{m,0}/3$ and *δ*_*m*,0_ is the Kronecker delta. In the above equations we drop the valley-specific coordinates in *f*_*n*,*m*,*μ*_ for notational simplicity, and the coordinates in $H_0^{(m)}$ and the right hand side are understood to belong to the valley of the envelope function that they act on.

**Fig. 1:**
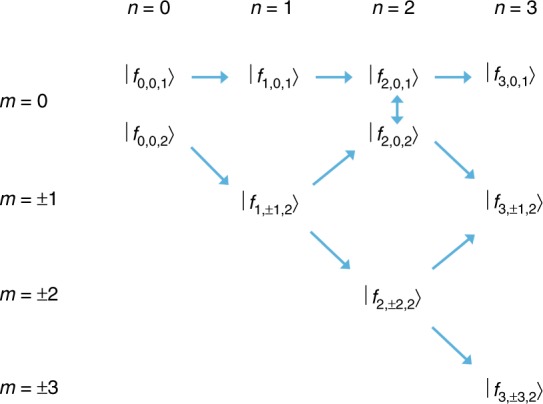
Multiphoton intermediate states *f*_*n*,*m*,*μ*_ and their interactions produced by dipole excitation polarized along *x*_1_ (horizontal arrows for the *μ* = 1 valley and diagonal arrows for the *μ* = 2 valley) and produced by *U*_*cc*_ (vertical arrows)

It is evident that Eq. (8) are not coupled by *U*_*cc*_ when the envelope function is zero at the origin. The ground state |*ψ*_0_〉 = |*ψ*_g_〉 has only *m* = 0 components, and it has even parity. Therefore, |*ψ*_1_〉 has odd parity according to Eq. (8), so the *U*_*cc*_ coupling term is suppressed. By the same logic, the *U*_*cc*_ coupling is only non-zero for even *n* and *m* = 0. In the case of $\left| {f_{n,m,1}} \right\rangle$, there is only dipole coupling to the functions with the same *m*, while for $\left| {f_{n,m,2}} \right\rangle$ the dipole coupling is to states with Δ*m* = ±1. The latter couplings are identical, so *f*_*n*,−*m*,*μ*_ = *f*_*n*,*m*,*μ*_. Figure 1 shows how the intermediate states are coupled by dipole excitation and the CCC.

**Fig. 2:**
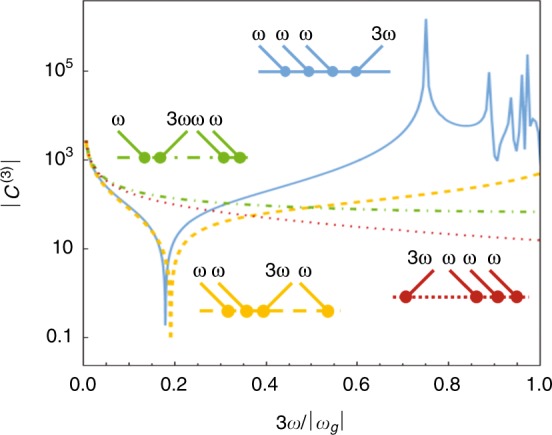
Contributions to *C*^(3)^ from the resonant and anti-resonant terms for hydrogen: 〈*ψ*_0_|*ζG*_3_*ζG*_2_*ζG*_1_*ζ*|*ψ*_0_〉 (blue), 〈*ψ*_0_|*ζG*_−1_*ζG*_2_*ζG*_1_*ζ*|*ψ*_0_〉 (yellow), 〈*ψ*_0_|*ζG*_−1_*ζG*_−2_*ζG*_1_*ζ*|*ψ*_0_〉 (green), and 〈*ψ*_0_|*ζG*_−1_*ζG*_−2_*ζG*_−3_*ζ*|*ψ*_0_〉 (red). At *ω* = 0, the two terms containing *G*_−2_ have opposite signs to the two terms with *G*_2_, and the sum tends to 222

**Fig. 3:**
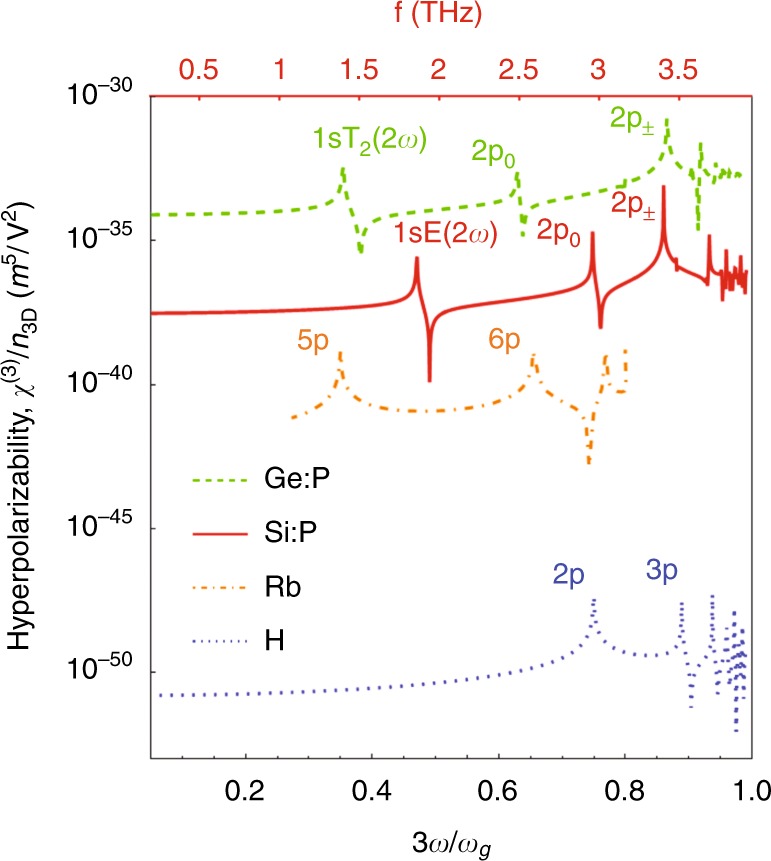
The *χ*^(3)^ spectrum for hydrogenic donors Si:P and Ge:P, with light polarized along a valley axis in each case, and hydrogen (all calculations from this work). A hydrogenic atomic vapor (Rb) is shown for comparison (data from ref. ^6^). Labels indicate the excited state for 3*ω* = *ω*_*eg*_ resonances and one 2*ω* resonance. The top axis applies only to Si:P and indicates the frequency in THz

Equation (8) can be solved by sequential application of the 2D FEM^15^. To test our numerical calculation we first compute *C*^(3)^ for hydrogen, and each of the resonant and antiresonant terms is shown in Fig. 2. Their sum is shown in Fig. 3, and we find excellent agreement within 0.2% of the previous result obtained from a Sturmian coulomb Green function in ref. ^16^.

## Discussion

### Giant third-order nonlinear susceptibility

**Fig. 4:**
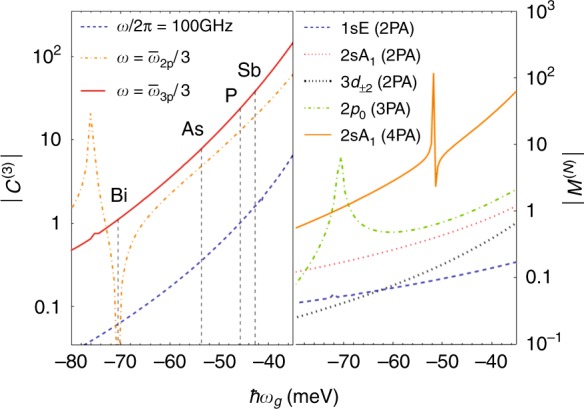
The effect of the CCC on *C*^(3)^ (left panel) and the NPA absorption matrix element *M*^(*N*)^ (right panel). The abscissa is the binding energy of the ground state (which is 31.5 meV at *U*_*cc*_ = 0), $\bar \omega _{2p} = (\omega _{2p_0} + \omega _{2p_ \pm })/2 - \omega_g$ is the average transition frequency to the 2*p* levels, and likewise for $\bar \omega _{3p}$. The binding energies of the Group V shallow donors are indicated in the left panel. The resonance and zero-crossing in the left panel, as well as the peaks in the 2*p*_0_ (3PA) and 2*s*A_1_ (4PA) matrix element on the right are due to the (2*ω*) resonance with the intermediate 1sE state

**Fig. 5:**
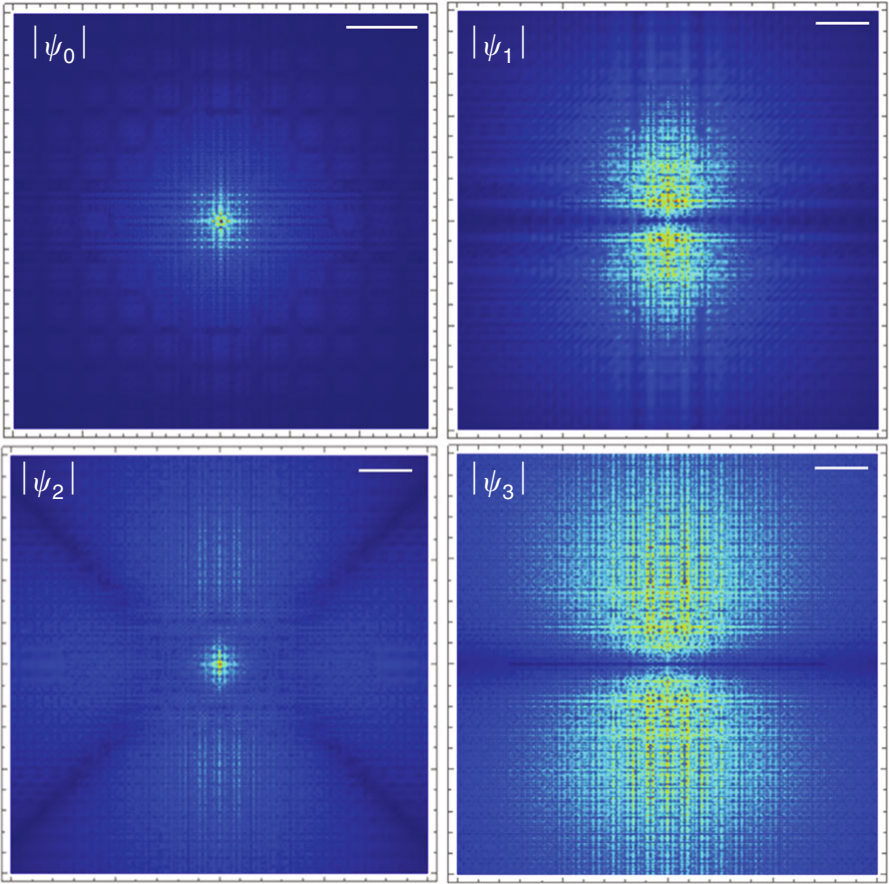
The wavefunctions |*ψ*_0_〉, |*ψ*_1_〉, |*ψ*_2_〉 and |*ψ*_3_〉 for Si:P (i.e., a binding energy of ℏω_g_ = −45.5 meV) in the x_3_ = 0 plane. The frequency used for this calculation is the average of the 2*p*_0_ and 2*p*_±_ resonances, and the color scale is normalized separately for each panel. The white bars on the top right indicate a length scale of 5 nm

Since silicon and germanium donors have an isotropic potential in an isotropic dielectric, the lowest-order nonlinear response is determined by *χ*^(3)^. The *χ*^(3)^ spectrum for each (including the antiresonant terms) is shown in Fig. 3. We took the parameters for silicon obtained from spectroscopic^17^ and magneto-optical measurements^12,18^, which are *γ* ≈ 0.208, *a*_*B*_ ≈ 3.17 nm and *E*_*H*_ ≈ 39.9 meV. The parameters for germanium are *γ* ≈ 0.0513, *a*_*B*_ ≈ 9.97 nm and *E*_*H*_ ≈ 9.40 meV^19^. Resonances occur when 3*ω* = *ω*_*eg*_, labeled according to |*ψ*_*e*_〉, and there are also sign-changes at which |*χ*^(3)^| goes to zero. In the range of frequency shown, we also observe a two-photon resonance for 1*s*A_1_ → 1*s*E, which is an obvious illustration of the need for a multivalley theory. There is no 3*ω* resonance with 1*s*T_2_ within the approximations made above in which there is no intervalley dipole coupling. The effect of *U*_*cc*_ on *χ*^(3)^ and the NPA matrix element is shown in Fig. 4. The low-frequency response of *C*^(3)^ is illustrated at 100 GHz. Two higher-frequency curves are included, with both far from 3*ω* resonances, half way between the 2*p*_0_ and 2*p*_±_ resonances, and between the 3*p*_0_ and 3*p*_±_. We choose these average frequencies since *χ*^(3)^ for Si:P varies slowly around them (see Fig. 3) and hence would not be sensitive to small experimental variations in the light frequency. For the 2*p*-average frequency, the 2*ω* resonance with the 1*s*E produces a coincidental zero-crossing for Si:Bi. Example results for the intermediate state wave functions produced in the calculation are shown in Fig. 5. The state |*ψ*_2_〉 is much larger in extent (and in magnitude) than |*ψ*_0_〉, and the extra node in the radial dependence due to the contribution of 2*s* is visible at about 5 nm. Similarly, the state |*ψ*_3_〉 is much larger in extent (and in magnitude) than |*ψ*_1_〉.

The square bracket in Eq. (5) gives the scaling of *χ*^(*N*)^ from hydrogenic atoms in vacuum to hydrogenic impurities in semiconductors, just as that in Eq. (1) does for *w*^(*N*)^, and as before, the much smaller *I*_*a*_ greatly increases the strength of the non-linearity. For example, the low-frequency limit of the hyperpolarizability *χ*^(3)^/*n*_3D_ for Si:P is much larger than that for hydrogen or alkali metal vapors such as Rb^6^, as shown in Fig. 3.

Some of the highest values of *χ*^(3)^ have been reported for solids, e.g., 2.8 × 10^−15^ m^2^/V^2^ for InSb^20^ and 2 × 10^−16^ m^2^/V^2^ for GaTe^21^. To convert the hyperpolarizability to a bulk *χ*^(3)^ value requires the concentration. To match InSb with Si:P at low frequency where *C*^(3)^ ≈ 1 (Fig. 4) (and *χ*^(3)^/*n*_3D_ = 2.9 × 10^−38^ m^5^/V^2^) requires a donor density of *n*_3D_ = 10^17^ cm^−3^ (where the donor–donor distance is 10*a*_*B*_). At high frequency, the hyperpolarizability is much higher, but the density should be lower to avoid inhomogeneous concentration broadening of the nearby excited levels. For example, *C*^(3)^ ≈ 20 between the 2*p*_0_ and 2*p*_±_ resonances at $\omega = \bar \omega _{2p}/3 = 2\pi \times 3.2\,{\text{THz}}$ (Fig. 4), and we match InSb at a density of *n*_3D_ = 5 × 10^15^ cm^−3^ at which concentration the 2p lines are well resolved^22^. If 3*ω* is moved even closer to the 2*p*_±_ resonance (or if the resonance is tuned with a magnetic field^18^), then *χ*^(3)^ could easily exceed InSb. Losses due to dephasing by phonon scattering may become important if the time spent in the intermediate states exceeds the phonon lifetime. Since the inverse of the former is given approximately by the detuning (Δ*f*Δ*t* ≥ 1/2*π*) and the inverse phonon-limited width (1/*πT*_2_ = 1 GHz^23,24^), then this loss is negligible for much of the spectrum. At 50 GHz below the 2*p*_±_ line so that such losses may be ignored, *C*^(3)^ ≈ 200, and *χ*^(3)^ is an order of magnitude above InSb.

We are not aware of any larger values for bulk media, but higher “bulk” values have been reported for 2D systems such as graphene and MoS_2_ for which *χ*^(3)^*L* data are divided by an interaction thickness *L* to obtain *χ*^(3)^; in particular, reports for graphene range from 10^−19^ ^25,26^ to 10^−15^ m^2^/V^2^ ^27^ for near-IR excitation and up to 10^−10^ m^2^/V^2^ in the THz region under resonant enhancement by landau levels in a magnetic field^28^. A recent experiment with single-layer graphene at room temperature reports a remarkably high value of 1.7 × 10^−9^ m^2^/V^2^ for the THz third-order nonlinear susceptibility^29^. In the case of coupled quantum wells (QW), large values of *χ*^(3)^ may be engineered through resonances, as demonstrated up to 10^−14^ m^2^/V^2^ ^30^. However, since the non-linear effect is limited by the interaction length, the 2D *χ*^(3)^*L* is probably a better figure of merit in these cases. For THz field-enhanced graphene with 50 layers, *χ*^(3)^*L* = 9 × 10^−20^ m^3^/V^2 ^^28^, and for single-layer graphene χ^(3)^*L* = 5.1 × 10^−19^ m^3^/V^2^ ^29^, or *χ*^(3)^*L* = 1.4 × 10^−18^ m^3^/V^2^ for resonant coupled QWs^30^. Even higher values are predicted for doped QWs up to *χ*^(3)^*L* = 5 × 10^−17^ m^3^/V^2 ^^31^. To match this value with Si:P at $\omega = \bar \omega _{2p}/3 = 2\pi \times 3.2\,{\text{THz}}$ and *n*_3D_ = 5 × 10^15^ cm^−3^ (see above) would require a sample thickness of *L* = 2 cm. Obviously, the required thickness can be significantly reduced when close to resonance, or for germanium.

### Efficient third-harmonic generation

The non-linear susceptibility is important for predicting the strength of frequency conversion processes such as third-harmonic generation (3HG), and we use this as an example application to investigate the utility of the medium. A solution for the amplitude of the generated wave produced by 3HG, neglecting absorption, is given by^32^. Converting to irradiance in MKS units,

9 $$\frac{{I_{{\mathrm{out}}}}}{{I_{{\mathrm{in}}}}} = \left( {\frac{{3\omega _{{\mathrm{in}}}\chi ^{(3)}LI_{{\mathrm{in}}}}}{{4\epsilon _0n^2c^2}}} \right)^2 = \left( {\frac{{I_{{\mathrm{in}}}f_{{\mathrm{in}}}n_{2{\mathrm{D}}}}}{x}C^{(3)}} \right)^2$$

where *I*_in_ is the irradiance of the input pump wave at frequency *f*_in_, and *n* is the geometric mean of the refractive indexes for the input and output waves, and *n*_2D_ = *n*_3D_*L*. Note that the isotropy mentioned earlier means that the polarization of the input and output waves must be parallel. We ignored a factor for the phase matching, which is unity if the length of the sample *L* ≪ *L*_*c*_, where the coherence length *L*_*c*_ = *πc*/(3*ω*_in_[*n*_out_ − *n*_in_]). Si:P at room temperature has a nearly constant *n* = 3.4153 in the range from 1 THz to 12 THz^33^, leading to typical values of *L*_*c*_ ≈ 10 cm. The factor *x* = 6.9 × 10^23^ W/cm^2^ × THz × cm^−2^ for silicon. For comparison, germanium has *x* = 9.2 × 10^19^ W/cm^2^ × THz × cm^−2^.

To illustrate the possible applications of this high *χ*^(*N*)^, we note that two types of THz diode lasers are available, the quantum cascade laser (QCL) from 0.74 THz^34^ to 5.4 THz^35^ with output powers of up to a few W^36,37^, and the hot hole (p-Ge) laser^38,39^ with a similar range and power. However, there is a large gap in the availability of solid-state sources from about 5 THz to about 12 THz^40^, where the GaAs Reststrahlen band renders laser operation impossible. This is an important region for quantum qubit applications^41–44^. Currently, the gap is only filled by larger, more expensive systems (difference frequency generators and free electron lasers). Tripling the output of 2–4 THz QCLs would fill the gap, but their output powers are far smaller than those typical for a pump laser in standard tripling applications, so a giant non-linearity is critical. At $\omega = \bar \omega _{2p}/3 = 2\pi \times 3.2\,{\text{THz}}$, *C*^(3)^ ≈ 20, so for *n*_2D_ = 10^16^ cm^−2^ (see above), a 1% predicted conversion may be obtained with 100 kW/cm^2^, and by moving to 50 GHz below the 2p_±_ resonance this value could be brought down to 10 kW/cm^2^, which is just about achievable with a well focussed QCL, and would thus provide enough output for spectroscopy applications. A nonlinear process that may possibly reduce the 3HG efficiency is multiphoton ionization^45^ since it reduces the population of the donors in the ground state. When $\omega = \bar \omega _{2p}/3$, for example, a four-photon absorption takes the electron to the continuum. We estimate this ionization in Si:P using the implicit summation method and find that the rate is *w* = 3.17 s^−1^ for *I*_in_ = 10 kW/cm^2^. This result simply means that the pulses must be kept significantly shorter than a second to avoid significant ionization.

In summary, we calculated the absolute values of the THz non-linear coefficients for the most common semiconductor materials, lightly doped silicon and germanium, which are available in the largest, purest and most regular single crystals known. The values we obtain for off-resonance rival the highest values obtained in any other material even when resonantly enhanced, and the material could gain new applications in THz photonics. We also predict the highly efficient third-harmonic generation of THz light in doped silicon and germanium. Our multi-valley theory for nonlinear optical processes of donors in silicon and germanium can be readily applied to any donor in any semiconductor host in which the effective mass approximation is reliable.

## Materials and methods

Details of the finite element computation used for solving the coupled partial differential equations (Eq. (8)) are provided in the Supplementary Material.

## Supplementary information

Supplementary material

## Data Availability

Data for Nguyen Le et al. Giant non-linear susceptibility of hydrogenic donors in silicon and germanium, 10.5281/zenodo.3269481. The data underlying this work is available without restriction.
