# Supplementary material for: Giant non-linear susceptibility of hydrogenic donors in silicon and germanium
Source: Light Sci Appl. 2019 Jul 10;8:64. doi: 10.1038/s41377-019-0174-6 (PMC6804565; doi:10.1038/s41377-019-0174-6)
Supplement: Supplementary file 1 — Supplementary material [file 41377_2019_174_MOESM1_ESM.pdf]

Supplementary materials for Nguyen Le et al "Giant non-linear susceptibility in silicon hydrogenic donors"

Nguyen H. Le,[1] Grigory V. Lanskii,[2] Gabriel Aeppli,[3,4,5] and Benedict N. Murdin[1]

[1]Advanced Technology Institute and SEPNet, University of Surrey, Guildford, GU2 7XH, United Kingdom
[2]Institute of Monitoring of Climatic and Ecological Systems SB RAS, 10/3 Academical Ave., Tomsk 634055, Russia
[3]Laboratory for Solid State Physics, ETH Zurich, Zurich, CH-8093,Switzerland
[4]Institut de Physique, EPF Lausanne, Lausanne, CH-1015, Switzerland
[5]Swiss Light Source, Paul Scherrer Institut, Villigen PSI, CH-5232, Switzerland

There are several theoretical innovations described in the main paper, and in this supplementary materials we provide the derivations. Implicit summation has already been developed for hydrogen, and several versions of the multivalley effective mass for silicon donors are available with different techniques and levels of approximation. Here we have developed:

1. The implicit summation technique for multiphoton non-linear susceptibility and multiphoton absorbtion in a multivalley donor, including an approximate central cell correction.

2. A successful but simple 2D Finite Element Method (FEM) for solving for the partial differential equations that appear in the implicit summation technique, including the inter-valley coupling due to the CCC, that can be written in a few lines of code in standard packages (we used Mathematica). The same method can be used to obtain the wavefunction and energy of a multivalley donor.

In order to implement the implicit summation technique for the multiphoton process we will require solution of the Schrödinger equation $H|\psi_j\rangle = \hbar\omega_j|\psi_j\rangle$ for the ground state and for the excited state in the case of absorption, and we will also require solution of PDEs of the form $[H - W_n]|\psi_n\rangle = \epsilon.\mathbf{r}|\psi_{n-1}\rangle$ where H is the Hamiltonian and $|\psi_0\rangle$ is the ground state. We therefore require a prescription for both diagonalising H and using it in a PDE, ideally consistently. In the main text we derive the Schrodinger equation and the PDEs for the intermediate states in the valley-specific spherical coordinates of the lab frame, in practice it is better to work in a stretched frame where the kinetic term is isotropic as the FEM converges faster with increasing mesh density in this frame (see below). In this Supplementary Materials all the equations are expressed in the spherical coordinates of this stretched frame.

I. MULTIVALLEY SCHRÖDINGER EQUATION

Silicon is indirect with six equivalent conduction band minima (valleys) near the Brillouin zone edge X-points, each characterized by Fermi surfaces that are prolate ellipsoids, characterized by the effective mass ratio γ. According to the Kohn-Luttinger effective mass approximation (EMA) [1], the wavefunction $\psi_j(\boldsymbol{r})$ of a shallow donor can be decomposed into six equivalent, slowly varying hydrogenic envelope functions, each modulated by plane wave functions corresponding to crystal momentum at the minima $\mathbf{k}_\mu$ (and a lattice periodic function that is unimportant here). The Hamiltonian comprises a kinetic energy term that operates only on the envelope function, the slowly varying Coulomb potential of the donor $V(\mathbf{r})$, and a rapidly varying potential due to the quantum defect that is short range, $U(\mathbf{r})$, referred to as the central cell correction (CCC). The kinetic energy has cylindrical symmetry because $\gamma \neq 1$, and $V(\mathbf{r})\&U(\mathbf{r})$ are spherical and tetrahedral respectively. The first two terms produce wavefunctions that are approximately hydrogen-like and the third mixes them to produce states that transform as the A_1, E and T_2 components of the T_d point group. We write

$$\psi_j(\boldsymbol{r}) = \sum_{\mu=\pm1,\pm2,\pm3} e^{i\mathbf{k}_\mu\cdot\mathbf{r}} u_{\boldsymbol{k}_\mu}(\boldsymbol{r}) F_{j,\mu}(\boldsymbol{r}), \tag{1}$$

where $F_{j,\mu}(\boldsymbol{r})$ is the envelope function and the valley index μ runs over $\pm1, \pm2, \pm3$ where 1,2,3 are the three crystal axes. For simplicity of derivation we first assume that $u_{\boldsymbol{k}_\mu}(\boldsymbol{r}) = 1$. A more rigorous derivation where this assumption is relaxed is provided in Sec. III. We note that there is no change in the form of the final equations.

Substituting Eqn (1) (with $u_{\boldsymbol{k}_\mu}(\boldsymbol{r}) = 1$) into the Schrödinger equation

$$H|\psi_j\rangle = \hbar\omega_j|\psi_j\rangle \tag{2}$$

within the EMA produces

$$\sum_{\mu=\pm1,\pm2,\pm3} e^{i\mathbf{k}_\mu\cdot\mathbf{r}} [H_0 + U(\boldsymbol{r}) - \hbar\omega_j] F_{j,\mu}(\boldsymbol{r}) = 0, \tag{3}$$

where

$$H_0 = -\frac{E_H a_B^2}{2}\left[\frac{\partial^2}{\partial x^2} + \frac{\partial^2}{\partial y^2} + \gamma\frac{\partial^2}{\partial z^2}\right] - \frac{E_H a_B}{\sqrt{x^2+y^2+z^2}}, \tag{4}$$

and the Coulomb potential $V(\boldsymbol{r})$ is the last term. Here $\gamma = m_t/m_l$, $a_B = 4\pi\epsilon_0\epsilon_r\hbar^2/m_t e^2$ and $E_H = m_t e^4/(4\pi\epsilon_0\epsilon_r)^2$ where m_t is the transverse effective mass, m_l the longitudinal effective mass and ϵ_r the dielectric constant of silicon. It has been written using a valley-specific coordinate system x, y, z where z is the valley axis, i.e. this frame is rotated relative to the lab frame x_1, x_2, x_3. In all this work x, y, z (and later r', θ', ϕ') refer to the valley coordinate frame and x_1, x_2, x_3 refer to the lab frame crystal axes.

The plane wave factors are quickly varying compared with every other factor except $U(\mathbf{r})$. Since $U(\boldsymbol{r})$ is very short-range, it effectively samples the wavefunction amplitude at the origin. Premultiplying Eqn (3) by $e^{-i\mathbf{k}_{\mu'}\cdot\mathbf{r}}$ and averaging over a volume $(2\pi/k_0)^3$ around $\mathbf{r}$ where $k_0 = |\mathbf{k}_\mu|$ produces

$$\left[H_0 - \hbar\omega_j\right] F_{j,\mu}(\boldsymbol{r}) + \sum_{\mu'=\pm1,\pm2,\pm3} U_{\mu\mu'}\delta(\boldsymbol{r})F_{j,\mu'}(\boldsymbol{r}) = 0, \tag{5}$$

where $\delta(\boldsymbol{r})$ is the Dirac delta function and

$$U_{\mu\mu'} = \int d\boldsymbol{r}\, e^{i(\boldsymbol{k}_{\mu'}-\boldsymbol{k}_\mu).\boldsymbol{r}}U(\boldsymbol{r}). \tag{6}$$

Clearly the U term only affects states with significant amplitude at $\mathbf{r} = 0$ due to the δ-function, i.e. principally the $1s$ state.

For an A_1 state all the envelope functions have the same amplitude at $r = 0$, hence $\sum_{\mu'} U_{\mu\mu'}\delta(\boldsymbol{r})F_{j,\mu'}(\boldsymbol{r}) = -U_{cc}\delta(\boldsymbol{r})F_{j,\mu}(\boldsymbol{r})$ where $U_{cc} = -\sum_{\mu'} U_{\mu\mu'}$. It is found experimentally that for E and T_2 states the CCC has rather small effect and so we neglect it. The eigenproblems for A_1 and for E, T_2 states are then respectively:

$$\left[H_0 - U_{cc}\delta(\mathbf{r}) - \hbar\omega_j\right] F_{j,\mu}(\mathbf{r}) = 0, \tag{7}$$

$$\left[H_0 - \hbar\omega_j\right] F_{j,\mu}(\mathbf{r}) = 0. \tag{8}$$

These equations are the same for all μ, and we solve them using a valley-specific coordinate system x, y, z where z is the valley axis. The states with odd parity envelopes have $F_{j,\mu}(0) = 0$, so the A_1, E and T_2 states are degenerate, whereas even parity envelopes have $F_{j,\mu}(0) \neq 0$ and the central cell correction lowers the energy of the A_1 states.

II. FINITE ELEMENT METHOD FOR HYDROGENIC DONORS WITH ANISOTROPIC MASS AND CENTRAL CELL CORRECTION

Equations (7) and (8) can be solved with a Finite Element Method (FEM). We first perform a sequence of transformations in order to achieve a combination of simplification of the problem and improving the rate of convergence as the FEM mesh density is increased. First we transform x, y, z to an anisotropic frame that symmetrizes the kinetic energy (with the side-effect that the Coulomb potential is made anisotropic):

$$\begin{aligned} x &= x', \\ y &= y', \\ z &= \sqrt{\gamma}z', \end{aligned} \tag{9}$$

(and obviously z' is still the valley axis, and x', y', z' are still rotated relative to the lab frame) producing

$$H_0^s = -\frac{E_H a_B^2}{2}\left[\frac{\partial^2}{\partial x'^2} + \frac{\partial^2}{\partial y'^2} + \frac{\partial^2}{\partial z'^2}\right] - \frac{E_H a_B}{\sqrt{x'^2+y'^2+\gamma z'^2}}. \tag{10}$$

We now transform to spherical polar coordinates to take advantage of the spherical symmetry of the kinetic energy term, and again because it improves the FEM convergence

$$\begin{aligned} x' &= r'\sin\theta'\cos\phi', \\ y' &= r'\sin\theta'\sin\phi', \\ z' &= r'\cos\theta', \end{aligned} \tag{11}$$

where θ' is the zenith angle away from z' and ϕ' is the azimuthal angle around z' away from x', and the symmetrized polar Hamiltonian is

$$H_0^{sp} = -\frac{E_H a_B^2}{2}\left[\frac{1}{r'^2}\frac{\partial}{\partial r'}\left(r'^2\frac{\partial}{\partial r'}\right) + \frac{1}{r'^2\sin^2\theta'}\frac{\partial^2}{\partial\phi'^2} + \frac{1}{r'^2\sin\theta'}\frac{\partial}{\partial\theta'}\left(\sin\theta'\frac{\partial}{\partial\theta'}\right)\right] - \frac{E_H a_B}{r'\sqrt{1-(1-\gamma)\cos^2\theta'}}. \tag{12}$$

We choose the wavefunction

$$F_{j,\mu}(\boldsymbol{r}) = e^{im\phi'} f_{j,m,\mu}(r',\theta') \equiv e^{im\phi'}\frac{1}{r'}Y_{j,m}(r',\theta'), \tag{13}$$

and there is no μ dependence on the RHS because all valleys are equivalent and the coordinates are valley-oriented. Since m is a good quantum number we now have a 2D problem and Eqns (7) and (8) become

$$\left[H_0^{sp2d} - (U_{cc}/\sqrt{\gamma})\delta(\boldsymbol{r'}) - \hbar\omega_j\right] Y_{j,m}(r',\theta') = 0$$
$$\left[H_0^{sp2d} - \hbar\omega_j\right] Y_{j,m}(r',\theta') = 0. \tag{14}$$

where the symmetrized polar 2D Hamiltonian is

$$H_0^{sp2d} = -\frac{E_H a_B^2}{2}\left[\frac{\partial^2}{\partial r'^2} - \frac{m^2}{r'^2\sin^2\theta'} + \frac{1}{r'^2\sin\theta'}\frac{\partial}{\partial\theta'}\left(\sin\theta'\frac{\partial}{\partial\theta'}\right)\right] - \frac{E_H a_B}{r'\sqrt{1-(1-\gamma)\cos^2\theta'}}. \tag{15}$$

Finally, we compress the radial scale to a tangent space, which transforms the semi-infinite $r\theta$ plane onto the finite rectangle $0 < \eta < \pi/2, 0 < \theta < \pi$, and again we find that it gives the improved convergence with increasing FEM mesh density:

$$r' = r_0 \tan\eta, \tag{16}$$

The constant r_0 is a scaling factor, and numerical experiments show that it should be chosen to be comparable to the radius of wavefunction of interest for an accurate result. Now we have

$$\left[H_0^{sp2dc} - (U_{cc}/\sqrt{\gamma})\delta(\boldsymbol{r'}) - \hbar\omega_j\right] y_{j,m}(\eta,\theta') = 0, \tag{17}$$

$$\left[H_0^{sp2dc} - \hbar\omega_j\right] y_{j,m}(\eta,\theta') = 0, \tag{18}$$

where the symmetrized polar 2D compressed Hamiltonian is

$$H_0^{sp2dc} = -\frac{E_H a_B^2}{2r_0^2}\left(\cos^2\eta\frac{\partial}{\partial\eta}\left(\cos^2\eta\frac{\partial}{\partial\eta}\right) - \frac{m^2\cot^2\eta}{\sin^2\theta'} + \frac{\cot^2\eta}{\sin\theta'}\frac{\partial}{\partial\theta'}\left(\sin\theta'\frac{\partial}{\partial\theta'}\right)\right) - \frac{E_H a_B r_0^{-1}\cot\eta}{\sqrt{1-(1-\gamma)\cos^2\theta'}}. \tag{19}$$

For our numerical calculation we model the Dirac delta function $\delta(\boldsymbol{r'})$ by a short range step function $\Theta(r_{cc} - r')/(4\pi r_{cc}^3/3) = \Theta(r_{cc} - r_0\tan\eta)/(4\pi r_{cc}^3/3)$ where $r_{cc} \ll a_B$. We fix $r_{cc} = 0.1a_B$ and use a bisection procedure to find the value of U_{cc} that reproduces the experimental energy of the $1sA_1$ state in Eqn (7).

The net transformation is

$$x = r_0\tan\eta\sin\theta'\cos\phi',$$
$$y = r_0\tan\eta\sin\theta'\sin\phi',$$
$$z = \sqrt{\gamma}r_0\tan\eta\cos\theta'. \tag{20}$$

The wavefunctions should be normalized with

$$Z = 2\pi r_0\sqrt{\gamma}\int_0^{\pi/2} d\eta\sec^2\eta\int_0^{\pi} d\theta'\sin\theta'|y_{j,m}(\eta,\theta')|^2. \tag{21}$$

We can classify the wavefunction $y_{j,m}(\eta,\theta')$ by parity p and the quantum number m, with m an integer. The functions with p,m both odd or both even satisfy the symmetric condition $y(\eta,\theta') = y(\eta,\pi-\theta')$, while the functions with p,m opposite parity satisfy the anti-symmetric condition $y(\eta,\theta') = -y(\eta,\pi-\theta')$. Therefore we need to solve for $y(r,\theta')$ only in the domain $0 \le \theta' \le \pi/2$. The boundary conditions for symmetric and anti-symmetric functions in θ' are

$$\Gamma_s : y(0,\theta') = y(\pi/2,\theta') = 0,$$
$$\Gamma_a : y(0,\theta') = y(\pi/2,\theta') = y(\eta,\pi/2) = 0. \tag{22}$$

In our numerical calculation we choose $\eta_{\max} = \pi/2.1$ to avoid divergence in the numerical integral of the multiphoton matrix elements.

III. INTERVALLEY COUPLED MULTIPHOTON PDES

A. Silicon

In this section we derive the intervalley coupled PDEs that appear in the implicit summation technique (Eqn M8 in the main text). After obtaining the ground state $|\psi_0\rangle = |\psi_g\rangle$ we want to solve the PDE

$$(H - W_n)\,|\psi_n\rangle = E_H\zeta\,|\psi_{n-1}\rangle\,, \tag{23}$$

where $\zeta = \boldsymbol{\epsilon}.\boldsymbol{r}/a_B$, and we use a similar expansion for the multivalley intermediate state wavefunctions as was used for the eigenstates of H in Sec. I.

$$\psi_n(\boldsymbol{r}) = \sum_\mu e^{i\mathbf{k}_\mu.\mathbf{r}} u_{\boldsymbol{k}_\mu}(\boldsymbol{r}) F_{n,\mu}(\boldsymbol{r}), \tag{24}$$

and $\psi_0(\boldsymbol{r})$ is given by the A_1 ground state, hence $F_{0,\mu}(\boldsymbol{r})$ can be obtained from solving Eqn (7). Again assuming $u_{\boldsymbol{k}_\mu}(\boldsymbol{r}) = 1$ as in Sec. I and substituting into Eqn (23), we obtain within the EMA

$$(H - W_n)\psi_n(\boldsymbol{r}) = \sum_\mu e^{i\mathbf{k}_\mu.\mathbf{r}}[H_0 + U(\boldsymbol{r}) - W_n]F_{n,\mu}(\boldsymbol{r}) = E_H\zeta \sum_\mu e^{i\mathbf{k}_\mu.\mathbf{r}} F_{n-1,\mu}(\boldsymbol{r}). \tag{25}$$

Premultiplying by $e^{-i\mathbf{k}_{\mu'}.\mathbf{r}}$ and averaging over a volume $(2\pi/k_0)^3$ around $\mathbf{r}$ produces

$$[H_0 - W_n]\,F_{n,\mu}(\boldsymbol{r}) + \sum_{\mu'} U_{\mu\mu'}\delta(\mathbf{r})F_{n,\mu'}(\boldsymbol{r}) = E_H\zeta F_{n-1,\mu}(\boldsymbol{r}), \tag{26}$$

where $U_{\mu\mu'}$ is given in Eqn (6). In this paper we neglect the energy shift due to the CCC of the 1sE and 1sT$_2$ states, and this is equivalent to assuming that $U_{\mu\mu'}$ is nearly the same for all μ, μ'. Let us denote $U_\parallel$ for $\mu' = \mu$, $U_\pm$ for $\mu' = -\mu$, and $U_\perp$ for all other combinations, in a perturbative treatment of the CCC the energy shift of the 1sA$_1$, 1sE and 1sT$_2$ are given by $(U_\parallel + U_\pm + 4U_\perp)|F_{\rm KL}(0)|^2$, $(U_\parallel + U_\pm - 2U_\perp)|F_{\rm KL}(0)|^2$, and $(U_\parallel - U_\pm)|F_{\rm KL}|(0)|^2$, respectively [2], where $F_{\rm KL}(\boldsymbol{r})$ is the unperturbed single-valley envelope function of the ground state without the CCC, as discussed by Kohn and Luttinger [1]. Neglecting the shift in the 1sE and 1sT$_2$ states means that

$$U_\parallel \approx U_\pm \approx U_\perp = -U_{cc}/6, \tag{27}$$

so we can replace $U_{\mu\mu'}$ by $-U_{cc}/6$. By symmetry (i.e knowing that we start from the symmetric A_1 ground state which has $|g,\mu\rangle$ the same for each valley with equal amplitudes, and knowing that the polarization ζ is the only factor that breaks the symmetry), $F_{n,\mu} = F_{n,-\mu}$, so each U_{cc} term is doubled up and there are three coupled equations:

$$\begin{aligned}
\left[H_0 - W_n - \frac{U_{cc}}{3}\delta(\mathbf{r})\right] F_{n,1}(\boldsymbol{r}) - \frac{U_{cc}}{3}\delta(\mathbf{r})\left[F_{n,2}(\boldsymbol{r}) + F_{n,3}(\boldsymbol{r})\right] &= E_H\zeta F_{n-1,1}(\boldsymbol{r}),\\
\left[H_0 - W_n - \frac{U_{cc}}{3}\delta(\mathbf{r})\right] F_{n,2}(\boldsymbol{r}) - \frac{U_{cc}}{3}\delta(\mathbf{r})\left[F_{n,1}(\boldsymbol{r}) + F_{n,3}(\boldsymbol{r})\right] &= E_H\zeta F_{n-1,2}(\boldsymbol{r}),\\
\left[H_0 - W_n - \frac{U_{cc}}{3}\delta(\mathbf{r})\right] F_{n,3}(\boldsymbol{r}) - \frac{U_{cc}}{3}\delta(\mathbf{r})\left[F_{n,1}(\boldsymbol{r}) + F_{n,2}(\boldsymbol{r})\right] &= E_H\zeta F_{n-1,3}(\boldsymbol{r}).
\end{aligned} \tag{28}$$

In the above equations the Hamiltonian H_0 is given by Eqn (4) with the valley-specific coordinates in H_0 understood to be belong to the valley of the envelope function that it acts on. We wish to transform to the same frame as used for the eigenstate calculation. In solving for the eigenstates we use wavefunctions like Eqn (13) in the valley-specific anisotropic spherical polar frame (r', θ', ϕ') so that $\theta'\&\phi'$ are relative to the valley axis:

$$F_{n,\mu}(\boldsymbol{r}) = \sum_m e^{im\phi'} f_{n,m,\mu}(r', \theta') \equiv \sum_m \frac{1}{r'} e^{im\phi'} Y_{n,m,\mu}(r', \theta'), \tag{29}$$

and substitute into the PDEs, premultiply by $e^{im'\phi'}$ and integrate over ϕ'. The operator ζ is polarization dependent, and the valley specific coordinates make it valley-specific too. In the case of [100] polarized light i.e. $a_B\zeta = x_1$, we have $a_B\zeta_1 = z = \sqrt{\gamma}r'\cos\theta', a_B\zeta_2 = x = r'\sin\theta'\cos\phi', a_B\zeta_3 = y = r'\sin\theta'\sin\phi'$, and we obtain

$$\begin{aligned}
\left[H_0 - W_n - \mathcal{D}\right] Y_{n,m,1} - \mathcal{D}(Y_{n,m,2} + Y_{n,m,3}) &= (E_H/a_B)\sqrt{\gamma}r'\cos\theta' Y_{n-1,m,1},\\
\left[H_0 - W_n - \mathcal{D}\right] Y_{n,m,2} - \mathcal{D}(Y_{n,m,1} + Y_{n,m,3}) &= (E_H/a_B)r'\sin\theta'\frac{1}{2}\left(Y_{n-1,m-1,2} + Y_{n-1,m+1,2}\right),\\
\left[H_0 - W_n - \mathcal{D}\right] Y_{n,m,3} - \mathcal{D}(Y_{n,m,1} + Y_{n,m,2}) &= (E_H/a_B)r'\sin\theta'\frac{1}{2i}\left(Y_{n-1,m-1,3} - Y_{n-1,m+1,3}\right),
\end{aligned} \tag{30}$$

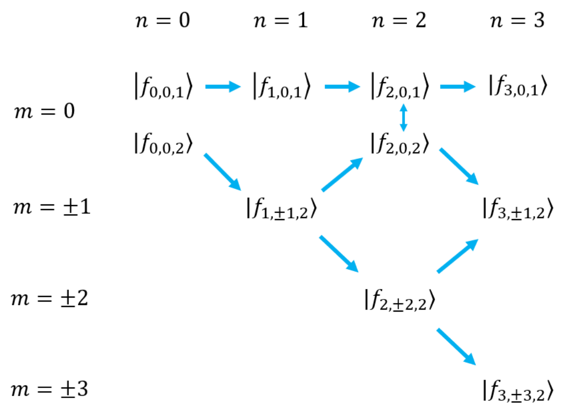

FIG. S1. Multiphoton intermediate states $|f_{n,m,\mu}\rangle$ and their interactions produced by dipole excitation along x_1 (horizontal arrows for the x_1 valley and diagonal arrows for the x_2 valleys) and produced by U_{cc} (vertical arrows). NB the x_3 valley function is the same as for the x_2 valley. For calculation of $\chi^{(N)}$ only $|f_{N,0,1}\rangle$ and $|f_{N,\pm1,2}\rangle$ are needed.

where H_0 is given in Eqn (15), $\mathcal{D} = (U_{cc}/3\sqrt{\gamma})\delta(\boldsymbol{r}')\delta_{m,0}$ and $\delta_{m,0}$ is the Kronecker Delta. In the above equations we drop the valley-specific coordinates in $Y_{n,m,\mu}$ for notational simplicity, and again the coordinates in H_0 and ζ belong to the valley of the envelope function that they act on. Note that the off-diagonal terms on the LHS are zero unless $m = 0$. The second and third equations are equivalent with

$$Y_{n,m,3} = i^{-m} Y_{n,m,2}, \tag{31}$$

so in the tangent space the $\Upsilon_{n,m,\mu}(\eta, \theta')$ are

$$[H_0 - W_n - \mathcal{D}]\,\Upsilon_{n,m,1} - 2\mathcal{D}\,\Upsilon_{n,m,2} = (E_H/a_B)\sqrt{\gamma} r_0 \tan\eta \cos\theta'\,\Upsilon_{n-1,m,1},$$
$$[H_0 - W_n - 2\mathcal{D}]\,\Upsilon_{n,m,2} + -\mathcal{D}\,\Upsilon_{n,m,1} = (E_H/a_B) r_0 \tan\eta \sin\theta' \frac{1}{2}\left(\Upsilon_{n-1,m-1,2} + \Upsilon_{n-1,m+1,2}\right),$$

where the Hamiltonian is now given by Eqn (19). It is evident that these equations are coupled by U_{cc} when $m = 0$ and the parity is even (shown by the vertical arrows on Fig. S1).

In the case of an arbitrary polarization $\boldsymbol{\epsilon}$, since we are free to rotate the valley-specific coordinates around the valley axis, we can always choose a coordinate such that $\boldsymbol{\epsilon}$ has only the components $\epsilon_\mu^{\parallel}$ along the z axis and $\epsilon_\mu^{\perp}$ along the x axis, and the component along y is zero, for *all* valleys μ. Repeating the steps for deriving Eqn (30) we obtain

$$[H_0 - W_n]\,Y_{n,m,\mu} - \mathcal{D}\sum_{\mu'} Y_{n,m,\mu'} = \frac{E_H}{a_B} r' \left[\epsilon_\mu^{\parallel}\sqrt{\gamma}\cos\theta' Y_{n-1,m,\mu} + \epsilon_\mu^{\perp}\frac{\sin\theta'}{2}\left(Y_{n-1,m-1,\mu} + Y_{n-1,m+1,\mu}\right)\right], \tag{32}$$

for $\mu, \mu' = 1, 2, 3$. By utilizing this free choice of coordinate rotation we see that the envelope functions depend only on the angle between the polarization vector and the valley axes. For polarization along the [1,1,1] crystal axis, for example, $\epsilon_\mu^{\parallel} = 1/\sqrt{3}$ and $\epsilon_\mu^{\perp} = \sqrt{2/3}$ for all μ, and it is evident from Eqn (32) that $Y_{n,m,\mu}$ must be the same for all μ, and Eqn (32) reduces to

$$[H_0 - W_n - 3\mathcal{D}]\,Y_{n,m,\mu} = \frac{E_H}{a_B} r' \left[\sqrt{\frac{\gamma}{3}}\cos\theta' Y_{n-1,m,\mu} + \sqrt{\frac{2}{3}}\frac{\sin\theta'}{2}\left(Y_{n-1,m-1,\mu} + Y_{n-1,m+1,\mu}\right)\right], \tag{33}$$

where $Y_{0,0,\mu} = Y_{g,0,\mu}$.

NB: Although the coordinate representation of vectors like $\boldsymbol{\epsilon}$ and functions like $Y_{n,m\mu}$ is coordinate dependent, scalars such as the NPA matrix element $M^{(N)}$ and the nonlinear susceptibility $C^{(N)}$ must be coordinate invariant. While one may obtain an apparent different form for $Y_{n,m,\mu}$ in a rotated frame, the final results for $M^{(N)}$ and $C^{(N)}$ have to stay the same.

B. Germanium

For germanium Eqn (32) also applies with $\mu, \mu' = 1, 2, 3, 4$ for the four CB minima valleys along the [1,1,1] equivalent crystal axes, and $\mathcal{D} = U_{cc}\delta(\boldsymbol{r}')\delta_{m,0}/4\sqrt{\gamma})$. The valley axes are given by the directional unit vectors $\boldsymbol{u}_\mu = [1, 1, 1]/\sqrt{3}$,

$[-1,1,1]/\sqrt{3}$, $[1,-1,1]/\sqrt{3}$ and $[1,1,-1]/\sqrt{3}$. For light polarized along the [1,1,1] crystal axis, the components of the polarization vector in the valley-specific coordinates are

$$\begin{aligned} \epsilon_1^{\|} &= \boldsymbol{\epsilon}.\boldsymbol{u}_1 = 1, \quad \epsilon_1^{\perp} = 0, \\ \epsilon_2^{\|} &= \boldsymbol{\epsilon}.\boldsymbol{u}_2 = 1/3, \quad \epsilon_2^{\perp} = \sqrt{1-(\epsilon_2^{\|})^2} = 2\sqrt{2}/3, \\ \epsilon_3^{\|} &= \boldsymbol{\epsilon}.\boldsymbol{u}_3 = 1/3, \quad \epsilon_3^{\perp} = 2\sqrt{2}/3, \\ \epsilon_4^{\|} &= \boldsymbol{\epsilon}.\boldsymbol{u}_4 = 1/3, \quad \epsilon_4^{\perp} = 2\sqrt{2}/3. \end{aligned} \tag{34}$$

Since $\boldsymbol{\epsilon}$ has the same components in valleys $2,3,4$, we see from Eqn (32) that $f_{n,m,2} = f_{n,m,3} = f_{n,m,4}$, and hence there are only two independent envelope functions, $f_{n,m,1}$ and $f_{n,m,2}$. Equation (32) now becomes

$$\begin{aligned} &\left[H_0^{(m)} - W_n - D\right] f_{n,m,1} - 3\mathcal{D} f_{n,m,2} = \frac{E_H}{a_B} r' \sqrt{\gamma}\cos\theta' f_{n-1,m,1}, \\ &\left[H_0^{(m)} - W_n - 3D\right] f_{n,m,2} - \mathcal{D} f_{n,m,1} = \frac{E_H}{a_B} r' \left[\frac{\sqrt{\gamma}\cos\theta'}{3} f_{n-1,m,2} + \frac{\sqrt{2}\sin\theta'}{3}\left(f_{n-1,m-1,2} + f_{n-1,m+1,2}\right)\right], \end{aligned} \tag{35}$$

We used the following parameters for Ge:P: $\gamma = 0.05134$, $E_H = 9.40$ meV, $a_B = 9.97$ nm [3], and $E(1s\mathrm{A}_1) = -12.89$ meV [4]. The interaction between the envelope functions $f_{n,m,\mu}$ for germanium is shown in Fig. S2.

IV. A RIGOROUS DERIVATION OF THE INTERVALLEY COUPLED MULTIPHOTON PDES

In the previous sections we derive the intervalley coupled multiphoton PDEs by assuming $u_{\boldsymbol{k}_\mu}(\boldsymbol{r}) = 1$, which allows an intuitive and simple derivation of the equations. Here we relax this assumption. We start with the full Hamiltonian including the silicon lattice potential, and approximations within the multivalley effective mass theory are stated clearly.

The Hamiltonian for an impurity electron is $H = H_{\mathrm{Si}} + V(r)$ where $H_{\mathrm{Si}} = -(\hbar^2/2m_e)\Delta + V_{\mathrm{Si}}(r)$ and $V_{\mathrm{Si}}(r)$ is the crystal potential of the silicon lattice. The impurity potential is $V(r) = -e^2/4\pi\epsilon_0\epsilon_r r + U(r)$, where $U(r)$ is the central cell correction.

The first Brillouin zone of silicon can be divided into six equal subregions Γ_μ, $\mu = \pm1, \pm2, \pm3$, each contains a conduction band minimum. In the one band approximation, the intermediate state $|\psi_n\rangle$ can be expanded using the Bloch states of the conduction band

$$\psi_n(\boldsymbol{r}) = \sum_\mu \sum_{\boldsymbol{k}\in\Gamma_\mu} F_{n,\mu}(\boldsymbol{k})\phi_{\boldsymbol{k}}(\boldsymbol{r}), \tag{36}$$

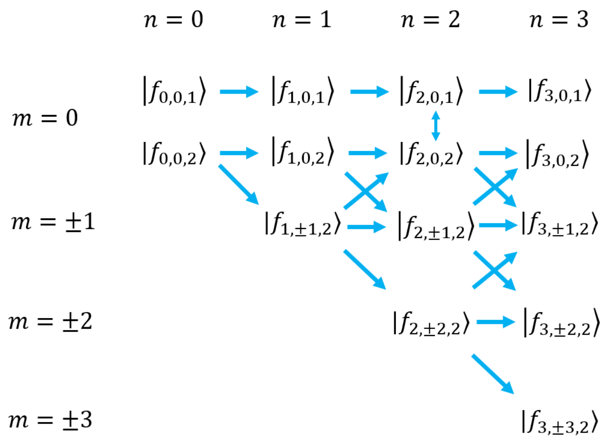

FIG. S2. Multiphoton intermediate states $|f_{n,m,\mu}\rangle$ and their interactions produced by dipole excitation (horizontal arrows and diagonal arrows) and produced by U_{cc} (vertical arrows) for germanium.

where $\phi_{\boldsymbol{k}}(\boldsymbol{r}) = e^{i\boldsymbol{k}.\boldsymbol{r}} u_{\boldsymbol{k}}(\boldsymbol{r})$ is the Bloch function of the pure crystal. It is the eigenstate of the unperturbed Hamiltonian

$$H_{\text{Si}}\phi_{\boldsymbol{k}}(\boldsymbol{r}) = E(\boldsymbol{k})\phi_{\boldsymbol{k}}(\boldsymbol{r}). \tag{37}$$

In the approximations of the multivalley effective mass theory, we assume $F_{n,\mu}(\boldsymbol{k})$ is non-vanishing only in a small pocket of the Brillouin zone around the conduction band minimum at $\boldsymbol{k}_\mu$, so we can replace $u_{\boldsymbol{k}}(\boldsymbol{r})$ by $u_{\boldsymbol{k}_\mu}(\boldsymbol{r})$ for each valley and Eqn (36) becomes

$$\psi_n(\boldsymbol{r}) = \sum_\mu F_{n,\mu}(\boldsymbol{r}) e^{i\boldsymbol{k}_\mu.\boldsymbol{r}} u_{\boldsymbol{k}_\mu}(\boldsymbol{r}), \tag{38}$$

where the envelope function is $F_{n,\mu}(\boldsymbol{r}) = \sum_{\boldsymbol{k}\in\Gamma_\mu} F_{n,\mu}(\boldsymbol{k}) e^{i(\boldsymbol{k}-\boldsymbol{k}_\mu).\boldsymbol{r}}$. The implicit-summation PDEs are

$$(H - W_n)\psi_n(\boldsymbol{r}) = \zeta(\boldsymbol{r})\psi_{n-1}(\boldsymbol{r}), \tag{39}$$

where $\zeta(\boldsymbol{r}) = \boldsymbol{\epsilon}.\boldsymbol{r}$. Substituting $\psi_n(\boldsymbol{r})$ from Eqn (36)

$$\sum_\mu \sum_{\boldsymbol{k}\in\Gamma_\mu} (E(\boldsymbol{k}) + V(r) - W_n) F_{n,\mu}(\boldsymbol{k})\phi_{\boldsymbol{k}}(\boldsymbol{r}) = \sum_\mu \sum_{\boldsymbol{k}\in\Gamma_\mu} \zeta(\boldsymbol{r}) F_{n-1,\mu}(\boldsymbol{k})\phi_{\boldsymbol{k}}(\boldsymbol{r}). \tag{40}$$

Multiply by $\phi^*_{\boldsymbol{k}'}(\boldsymbol{r})$ with $\boldsymbol{k}'$ from the valley Γ_ν, integrate over $\boldsymbol{r}$ and use the orthonormality of the Bloch functions, we have

$$(E(\boldsymbol{k}') - W_n)F_{n,\nu}(\boldsymbol{k}') + \sum_\mu \sum_{\boldsymbol{k}\in\Gamma_\mu} F_{n,\mu}(\boldsymbol{k}) \int d\boldsymbol{r}\, \phi^*_{\boldsymbol{k}'}(\boldsymbol{r}) V(r) \phi_{\boldsymbol{k}}(\boldsymbol{r}) = \sum_\mu \sum_{\boldsymbol{k}\in\Gamma_\mu} F_{n-1,\mu}(\boldsymbol{k}) \int d\boldsymbol{r}\, \phi^*_{\boldsymbol{k}'}(\boldsymbol{r})\zeta(\boldsymbol{r})\phi_{\boldsymbol{k}}(\boldsymbol{r}) \tag{41}$$

for $\boldsymbol{k}'$ closed to k_ν and $\boldsymbol{k}$ closed to k_μ, we apply the usual approximation of multivalley EMT and replace the atomic parts $u_{\boldsymbol{k}'}(\boldsymbol{r})$ and $u_{\boldsymbol{k}}(\boldsymbol{r})$ in the Bloch functions by $u_{\boldsymbol{k}_\nu}(\boldsymbol{r})$ and $u_{\boldsymbol{k}_\mu}(\boldsymbol{r})$, respectively. The above equation becomes

$$\begin{aligned}
(E(\boldsymbol{k}') - W_n)F_{n,\nu}(\boldsymbol{k}') &+ \sum_\mu \int d\boldsymbol{r}\, e^{-i(\boldsymbol{k}'-\boldsymbol{k}_\nu).\boldsymbol{r}} \left(\sum_{\boldsymbol{k}\in\Gamma_\mu} F_{n,\mu}(\boldsymbol{k}) e^{i(\boldsymbol{k}-\boldsymbol{k}_\mu).\boldsymbol{r}} \right) V_{\nu\mu}(\boldsymbol{r}) \\
&= \sum_\mu \int d\boldsymbol{r}\, e^{-i(\boldsymbol{k}'-\boldsymbol{k}_\nu.\boldsymbol{r})} \left(\sum_{\boldsymbol{k}\in\Gamma_\mu} F_{n-1,\mu}(\boldsymbol{k}) e^{i(\boldsymbol{k}-\boldsymbol{k}_\mu).\boldsymbol{r}} \right) \zeta_{\nu\mu}(\boldsymbol{r}), \\
(E(\boldsymbol{k}') - W_n)F_{n,\nu}(\boldsymbol{k}') &+ \sum_\mu \int d\boldsymbol{r}\, e^{-i(\boldsymbol{k}'-\boldsymbol{k}_\nu).\boldsymbol{r}} F_{n,\mu}(\boldsymbol{r}) V_{\nu\mu}(\boldsymbol{r}) \\
&= \sum_\mu \int d\boldsymbol{r}\, e^{-i(\boldsymbol{k}'-\boldsymbol{k}_\nu).\boldsymbol{r}} F_{n-1,\mu}(\boldsymbol{r})\zeta_{\nu\mu}(\boldsymbol{r}),
\end{aligned} \tag{42}$$

where the valley-orbit coupling potential and dipole operator are

$$\begin{aligned}
V_{\nu\mu}(\boldsymbol{r}) &= \phi^*_{\boldsymbol{k}_\nu}(\boldsymbol{r}) V(\boldsymbol{r}) \phi_{\boldsymbol{k}_\mu}(\boldsymbol{r}) \\
\zeta_{\nu\mu} &= \phi^*_{\boldsymbol{k}_\nu}(\boldsymbol{r}) \zeta(\boldsymbol{r}) \phi_{\boldsymbol{k}_\mu}(\boldsymbol{r}).
\end{aligned} \tag{43}$$

Multiply Eqn (42) by $e^{i(\boldsymbol{k}'-\boldsymbol{k}_\nu).\boldsymbol{r}'}$ and summing over $\boldsymbol{k}' \in \Gamma_\nu$ we have for each term

$$\begin{aligned}
\sum_{\boldsymbol{k}'\in\Gamma_\nu} (E(\boldsymbol{k}') - W_n)F_{n,\nu}(\boldsymbol{k}') e^{i(\boldsymbol{k}'-\boldsymbol{k}_\nu).\boldsymbol{r}'} &= \sum_{\boldsymbol{k}'\in\Gamma_\nu} (E(-i\boldsymbol{\nabla}' + \boldsymbol{k}_\nu) - W_n) F_{n,\nu}(\boldsymbol{k}') e^{i(\boldsymbol{k}'-\boldsymbol{k}_\nu).\boldsymbol{r}'} \\
&= (E(-i\boldsymbol{\nabla}' + \boldsymbol{k}_\nu) - W_n) F_{n,\nu}(\boldsymbol{r}'), \\
\sum_{\boldsymbol{k}'\in\Gamma_\nu} \sum_\mu \int d\boldsymbol{r}\, e^{-i(\boldsymbol{k}'-\boldsymbol{k}_\nu).(\boldsymbol{r}-\boldsymbol{r}')} F_{n,\mu}(\boldsymbol{r}) V_{\nu\mu}(\boldsymbol{r}) &= \sum_\mu \int d\boldsymbol{r} \left[\sum_{\boldsymbol{k}'\in\Gamma_\nu} e^{-i(\boldsymbol{k}'-\boldsymbol{k}_\nu).(\boldsymbol{r}-\boldsymbol{r}')} \right] F_{n,\mu}(\boldsymbol{r}) V_{\nu\mu}(\boldsymbol{r}) \\
&\approx \sum_\mu \int d\boldsymbol{r}\, \delta(\boldsymbol{r}-\boldsymbol{r}') F_{n,\mu}(\boldsymbol{r}) V_{\nu\mu}(\boldsymbol{r}) = \sum_\mu F_{n,\mu}(\boldsymbol{r}') V_{\nu\mu}(\boldsymbol{r}'), \\
\sum_{\boldsymbol{k}'\in\Gamma_\nu} \sum_\mu \int d\boldsymbol{r}\, e^{-i(\boldsymbol{k}'-\boldsymbol{k}_\nu).(\boldsymbol{r}-\boldsymbol{r}')} F_{n-1,\mu}(\boldsymbol{r}) \zeta_{\nu\mu}(\boldsymbol{r}) &\approx \sum_\mu F_{n-1,\mu}(\boldsymbol{r}') \zeta_{\nu\mu}(\boldsymbol{r}'),
\end{aligned} \tag{44}$$

and we arrive at the multivalley coupled equations for the envelope functions

$$(E(-i\boldsymbol{\nabla}+\boldsymbol{k}_\nu)-W_n)F_{n,\nu}(\boldsymbol{r})+\sum_\mu F_{n,\mu}(\boldsymbol{r})V_{\nu\mu}(\boldsymbol{r})=\sum_\mu F_{n-1,\mu}(\boldsymbol{r})\zeta_{\nu\mu}(\boldsymbol{r}). \tag{45}$$

Within the parabolic approximation for the conduction band minimum

$$E(-i\boldsymbol{\nabla}+\boldsymbol{k}_\nu)\approx-\frac{\hbar^2}{2m_t}\left(\frac{\partial^2}{\partial x_\nu^2}+\frac{\partial^2}{\partial y_\nu^2}\right)-\frac{\hbar^2}{2m_l}\frac{\partial^2}{\partial z_\nu^2}. \tag{46}$$

If we further neglect the inter-valley coupling due to the long range part $-e^2/(4\pi\epsilon_0\epsilon_r r)$ of the impurity potential and the inter-valley coupling in the dipole matrix element, and use $\phi^*_{\boldsymbol{k}_\nu}(\boldsymbol{r})\phi_{\boldsymbol{k}_\nu}(\boldsymbol{r})\approx 1$ we have

$$\left[-\frac{\hbar^2}{2m_t}\left(\frac{\partial^2}{\partial x_\nu^2}+\frac{\partial^2}{\partial y_\nu^2}\right)-\frac{\hbar^2}{2m_l}\frac{\partial^2}{\partial z_\nu^2}-\frac{e^2}{4\pi\epsilon_0\epsilon_r r}-W_n\right]F_{n,\nu}(\boldsymbol{r})+\sum_\mu U_{\nu\mu}(\boldsymbol{r})F_{n,\mu}(\boldsymbol{r})=\zeta(\boldsymbol{r})F_{n-1,\mu}(\boldsymbol{r}), \tag{47}$$

where

$$U_{\nu\mu}(\boldsymbol{r})=\phi^*_{\boldsymbol{k}_\nu}(\boldsymbol{r})U(\boldsymbol{r})\phi_{\boldsymbol{k}_\mu}(\boldsymbol{r}). \tag{48}$$

If $U(\boldsymbol{r})$ is very short range $U_{\nu\mu}(\boldsymbol{r})$ effectively samples the slow-varying envelope function at $r=0$, hence it is a good approximation to replace $U_{\nu\mu}(\boldsymbol{r})$ by $U_{\nu\mu}\delta(\boldsymbol{r})$ where

$$U_{\nu\mu}=\int d\boldsymbol{r}\phi^*_{\boldsymbol{k}_\nu}(\boldsymbol{r})U(\boldsymbol{r})\phi_{\boldsymbol{k}_\mu}(\boldsymbol{r}), \tag{49}$$

and we arrive at the same equation as Eqn (26) in Sec. III with the only difference being the form of $U_{\nu,\mu}$ which now has a contribution from $u_{\boldsymbol{k}_\mu}(\boldsymbol{r})$.

To obtain the multivalley Schrodinger equation discussed in Sec. I we replace in Eqn (39) $|\psi_n\rangle$ by $|\psi_j\rangle$, W_n by $\hbar\omega_j$ and set $\zeta(\boldsymbol{r})=0$. Repeating the same steps we arrive at

$$\left[-\frac{\hbar^2}{2m_t}\left(\frac{\partial^2}{\partial x_\nu^2}+\frac{\partial^2}{\partial y_\nu^2}\right)-\frac{\hbar^2}{2m_l}\frac{\partial^2}{\partial z_\nu^2}-\frac{e^2}{4\pi\epsilon_0\epsilon_r r}-\hbar\omega_j\right]F_{j,\nu}(\boldsymbol{r})+\sum_\mu U_{\nu\mu}\delta(\boldsymbol{r})F_{j,\mu}(\boldsymbol{r})=0, \tag{50}$$

which is the same as Eqn (26) but with a different form for $U_{\nu\mu}$. This is the Shindo-Nara multivalley efffective mass equation of Ref. [5].

V. MULTIVALLEY N-PHOTON ABSORPTION

The multi photon matrix element is produced by successive applications of the operators G_n and ζ, both of which mix m states. Since G_n contains H which contains U_{cc}, it produces inter-valley mixing (while H_0 and ζ do not). Consider the excitation from $|\psi_g\rangle\equiv|\psi_0\rangle$ to $|\psi_e\rangle$

$$\begin{aligned}M^{(N)}&=\langle\psi_e|\,\zeta G_{N-1}\zeta\ldots G_2\zeta G_1\zeta\,|\psi_g\rangle\\&=\langle\psi_e|\,\zeta\,|\psi_{N-1}\rangle\,.\end{aligned}$$

Now, often the excited state is part of a degenerate manifold, e.g. in our Si:P situation the states with m and $-m$ are degenerate. Also, the multivalley states with odd parity envelope have zero amplitude at $r=0$, so the CCC can also be neglected and the A_1, E and T_2 states are degenerate. At the same time, the intermediate state $\zeta\,|\psi_{N-1}\rangle$ also has multiple components. Let the set of degenerate components of the excited state be ψ_e which is a subset of the complete set of eigenstates and let the remainder be ψ_d, so that we may express the intermediate state as

$$\zeta\,|\psi_{N-1}\rangle=\sum_e\alpha_e\,|\psi_e\rangle+\sum_d\alpha_d\,|\psi_d\rangle$$

where $\alpha_j=\langle\psi_j|\zeta|\psi_{N-1}\rangle$. The excited state superposition that has greatest overlap with this state is

$$|\Psi_e\rangle=\frac{\sum_e\alpha_e\,|\psi_e\rangle}{\sqrt{\sum_e|\alpha_e|^2}},$$

and the matrix element is then

$$M^{(N)} = \frac{\sum_e \alpha_e \langle\psi_e|}{\sqrt{\sum_e |\alpha_e|^2}} \sum_e{}' \alpha_e' |\psi_e'\rangle = \sqrt{\sum_e |\alpha_e|^2},$$

$$|M^{(N)}|^2 = \sum_e | \langle\psi_e| \zeta |\psi_{N-1}\rangle |^2, \tag{51}$$

so the N-photon transition rate is the total transition rate to the degenerate manifold as it should be.

Resolving $|\psi_{N-1}\rangle$ into valley and azimuthal components (combining Eqns (24) and (29)

$$\psi_{N-1}(\boldsymbol{r}) = \sum_{m,\mu} e^{i\mathbf{k}_\mu\cdot\mathbf{r}} e^{im\phi} f_{N-1,m,\mu}(\boldsymbol{r}),$$

and similarly for

$$\psi_e(\boldsymbol{r}) = \sum_{\mu} e^{i\mathbf{k}_\mu\cdot\mathbf{r}} e^{im\phi} f_{e,m,\mu}(\boldsymbol{r}),$$

and

$$\zeta = \sum_{\Delta m=-1,0,1} \zeta_{\Delta m,\mu} e^{i\Delta m\phi}. \tag{52}$$

Calculating the matrix element by averaging over a volume $(2\pi/k_0)^3$ and integrating over ϕ as usual,

$$\langle\psi_e| \zeta |\psi_{N-1}\rangle = \sum_{\mu,\Delta m=-1,0,1} \langle f_{e,m+\Delta m,\mu}| \zeta_{\Delta m,\mu} |f_{N-1,m,\mu}\rangle . \tag{53}$$

VI. NON-LINEAR SUSCEPTIBILITY

The linear susceptibility (Eqn 3.2.23 in Ref. [6]) for polarization along axis x_ν produced by light polarized along $x_{\nu'}$ is

$$\chi^{(1)}(\omega) = \frac{n_{3D}e^2}{\epsilon_0\hbar} \sum_j \langle\psi_g| \zeta |j\rangle \langle j| \zeta |\psi_g\rangle \left[\frac{1}{(\omega_{jg}-\omega)} + \frac{1}{(\omega_{jg}+\omega)}\right], \tag{54}$$

where e is the electron charge and n_{3D} is the density of atoms, and the frequency ω is far from any resonances. For an incoming wave with just one polarization and frequency the second term in the bracket is "antiresonant."

$$\chi^{(1)}(\omega) = \frac{n_{3D}e^2}{\epsilon_0\hbar} \sum_j \frac{\langle\psi_g| \zeta |j\rangle \langle j| \zeta |\psi_g\rangle}{(\omega_{jg}-\omega)} = \frac{n_{3D}e^2 a_B^2}{\epsilon_0 E_H} C^{(1)}(\omega), \tag{55}$$

where

$$C^{(1)}(\omega) = \langle\psi_g| \zeta G_1 \zeta |\psi_g\rangle . \tag{56}$$

The second order susceptibility is zero because it contains terms like $\langle\psi_g| \zeta G_2 \zeta G_1 \zeta |\psi_g\rangle$ which are forbidden by parity since ζ is odd. The third order susceptibility is (Eqn 3.2.37 in Ref. [6])

$$\chi^{(3)}(3\omega) = \frac{n_{3D}(ea_B)^4}{\epsilon_0\hbar^3} \sum_{l,k,j} \langle\psi_g| \zeta |l\rangle \langle l| \zeta |k\rangle \langle k| \zeta |j\rangle \langle j| \zeta |\psi_g\rangle \tag{57}$$

$$\left[\frac{1}{(\omega_{lg}-3\omega)(\omega_{kg}-2\omega)(\omega_{jg}-\omega)} + \frac{1}{(\omega_{lg}+\omega)(\omega_{kg}-2\omega)(\omega_{jg}-\omega)} + \frac{1}{(\omega_{lg}+\omega)(\omega_{kg}+2\omega)(\omega_{jg}-\omega)} + \frac{1}{(\omega_{lg}+\omega)(\omega_{kg}+2\omega)(\omega_{jg}+3\omega)}\right],$$

where the first term in the bracket is the resonant term, and the other three are "antiresonant".

$$\chi^{(3)}(3\omega) = \frac{n_{3D}(ea_B)^4}{\epsilon_0 E_H^3} C^{(3)}(3\omega), \tag{58}$$

where

$$C^{(3)}(3\omega) = \langle\psi_g|\, \zeta G_3 \zeta G_2 \zeta G_1 \zeta \,|\psi_g\rangle = \langle\psi_g|\, \zeta \,|\psi_3\rangle \,, \tag{59}$$

for the resonant term. The other terms are produced by simple changes in G_n.

The resonant part of the N-th order susceptibility is

$$\chi^{(N)}(N\omega) = \frac{n_{3D}(ea_B)^{N+1}}{\epsilon_0 E_H^N} C^{(N)}(N\omega), \tag{60}$$

where

$$C^{(N)}(N\omega) = \langle\psi_g|\, \zeta G_n \ldots \zeta G_2 \zeta G_1 \zeta \,|\psi_g\rangle = \langle\psi_g|\, \zeta \,|N\rangle \,. \tag{61}$$

Clearly, extraction of the non-linear susceptibility requires calculation of a matrix element that is very similar to that for the multiphoton absorption involving the same PDEs, except that now $\omega \neq \omega_{eg}/N$. The final integral for the matrix element can be made in the same way as for $M^{(N)}$ with Eqn (53).

VII. THIRD HARMONIC GENERATION

The susceptibility calculated in the previous section is used for the prediction of non-linear optical processes. Here we give definitions to allow cross-comparison of the strength of one resulting effect, third harmonic generation (3HG) in hydrogenic donors in silicon and hydrogenic atoms in a gas.

External electric fields can polarize a medium, and if it is non-linear this can lead to the appearance of new frequency components, different from the external drive frequency. Since Si:P has an isotropic potential in an isotropic dielectric, its nonlinear response is determined to lowest order by $\chi^{(3)}$, and the corresponding third order polarization $\vec{P}^{(3)}$ has the form:

$$\vec{P}^{(3)} = \epsilon_0 \chi^{(3)} \vec{E}^3, \tag{62}$$

where ϵ_0 is the permittivity of free space and the drive electric field has amplitude $\vec{E}$ with single frequency component and propagating along z axis for simplicity

$$E(z,t) = E(z)e^{-i\omega_p t} + \text{c.c.}, \tag{63}$$

where $E(z)$ is the complex amplitude of the electric field:

$$E(z) = A_p(z)e^{-ik_p z}. \tag{64}$$

Using Eqn (62) one can obtain the polarization induced by the applied electric field

$$P^{(3)} = \epsilon_0 \chi^{(3)} \left(E(z)e^{-i\omega_p t} + E^*(z)e^{i\omega_p t}\right)^3 = \epsilon_0 \chi^{(3)} E^3(z) e^{-i3\omega_p t} + 3\epsilon_0 \chi^{(3)} E^2(z) E^*(z) e^{-i\omega_p t} + \text{c.c.} \tag{65}$$

The first term on the right hand side of this equation leads to third harmonic generation; and one can represent the third order polarization at the triple frequency ($\omega_o = 3\omega_p$) as

$$P^{(3)}_{3\omega_p}(z,t) = \epsilon_0 \chi^{(3)} A_p^3(z) e^{i3k_p z} e^{-i3\omega_p t}. \tag{66}$$

Wave coupling in nonlinear polarized media results in energy transfer between interacting waves. The wave equation for isotropic, nonmagnetic, dielectric media with nonlinear polarization $\vec{P}_{\text{NL}}$ results straightforwardly from the Maxwell equations and has the form:

$$\nabla^2 \vec{E} = \frac{\varepsilon_r}{c^2} \frac{\partial^2}{\partial t^2} \vec{E} - \frac{1}{\epsilon_0 c^2} \frac{\partial^2}{\partial t^2} \vec{P}_{\text{NL}}, \tag{67}$$

where ε_r is the relative dielectric permittivity. In the case of isotropic media ε_r is a scalar quantity and the (linear) refraction index $n^2 = \varepsilon_r$. We suppose that this equation is satisfied for each frequency component separately. Let's obtain derivatives of Eqn (67) for the output ($3\omega_p$) frequency component explicitly.

$$\frac{\partial^2}{\partial t^2} P_{3\omega_p} = -\left(3\omega_p\right)^2 \epsilon_0 \chi^{(3)} A_p^3(z) e^{i3k_p z} e^{-i3\omega_p t}, \tag{68}$$

$$\frac{\partial^2}{\partial t^2} E_{3\omega_p}(z,t) = -A_o(z)\omega_o^2 e^{ik_o z} e^{-i\omega_o t}, \tag{69}$$

$$\frac{\partial^2}{\partial z^2} E_{3\omega_p}(z,t) = e^{ik_o z} e^{-i\omega_o t} \left(\frac{\partial^2}{\partial z^2} A_o(z) + 2ik_o \frac{\partial}{\partial z} A_o(z) - A_o(z) k_o^2 \right), \tag{70}$$

where we can neglect the first item in the parenthesis as we suppose that the complex amplitude of the field varies slowly along z:

$$k\frac{\partial}{\partial z} A_o(z) \gg \frac{\partial^2}{\partial z^2} A_o(z).$$

Putting Eqns (68), (69), (70) into (67) one can get

$$\left(2ik_o \frac{\partial}{\partial z} A_o(z) - A_o(z) k_o^2\right) e^{ik_o z} e^{-i\omega_o t} + \frac{\varepsilon_r(3\omega_p)}{c^2} A_o(z)\omega_o^2 e^{ik_o z} e^{-i\omega_o t} = -\frac{1}{c^2}\left(3\omega_p\right)^2 \chi^{(3)} A_p^3(z) e^{i3k_p z} e^{-i3\omega_p t} \tag{71}$$

Inserting $\varepsilon_r/c^2 = k^2/\omega^2$, $3\omega_p = \omega_o$ and introducing the wavevector mismatch $\Delta k = 3k_p - k_o$, Eqn (71) becomes

$$\frac{\partial A_o(z)}{\partial z} = \frac{i}{2}\frac{\omega_o}{cn_o}\chi^{(3)} A_p^3(z) e^{i\Delta k z}. \tag{72}$$

This result is the so-called coupled-amplitude equation because it shows how the amplitude of the output wave depends on the three amplitudes of the pumping waves and the phase mismatch between them. It should to be noted that we can deduce similar equations for each of the interacting pump waves, but it is already clear that all amplitudes are functions of each other and there is no simple solution. However, if we assume that conversion of the pump power into the generated wave is small, i.e. the amplitude of the pumping wave is a constant along the nonlinear medium, then we can integrate Eqn (72):

$$A_o(z) = \frac{i}{2}\frac{\omega_o}{cn_o}\chi^{(3)} A_p^3 \int_0^L e^{i\Delta k z}\, dz = -\frac{\omega_o}{2cn_o}\chi^{(3)} A_p^3 \left(\frac{1 - e^{i\Delta k L}}{\Delta k}\right), \tag{73}$$

where L is the length of nonlinear medium. When $L = L_c = \pi/\Delta k$, the term in the parentheses of Eqn (73) reaches its maximum value of $2/\Delta k$. The length L_c is the so-called the coherence length, and above it power is converted backwards, from the third harmonic to the pump. Thus it limits the useful length of a media. For normal dispersion $\Delta k > 0$ in isotropic materials, but fortunately in the THz region a frequency dependence of the refraction is weak enough that the coherent length be as much as some tens centimeters.

Now we are ready to obtain an intensity of the output beam:

$$I_o = 2n_o\epsilon_0 c A_o A_o^* = 2n_o\epsilon_0 c \frac{\omega_o^2}{4c^2 n_o^2 \Delta k^2}\left(\chi^{(3)}\right)^2 A_p^6 \left(1 - e^{i\Delta k L}\right)\left(1 - e^{-i\Delta k L}\right). \tag{74}$$

Introducing parameter $x = \Delta k L/2$ the product of the final pair of brackets is $4(\sin x)^2$ and finally we get

$$I_o = \frac{\omega_o^2 L^2 I_p^3}{16c^4\epsilon_0^2 n_o n_p^3}\left(\chi^{(3)}\right)^2 \left(\frac{\sin x}{x}\right)^2. \tag{75}$$

One can represent this equation over the beam powers P in W and pump frequency ν_p in Hz:

$$P_o = \frac{36\nu_p^2 L^2 P_p^3}{c^4\epsilon_0^2 n_o n_p^3 d^4}\left(\chi^{(3)}\right)^2 \left(\frac{\sin x}{x}\right)^2, \tag{76}$$

where d is the beam diameter and all parameters have MKS dimensions.This expression for plane waves [7, 8] may be modified for 3HG in a gaussian beam [9, 10]. Equation (76) shows that the output power is proportional to the cube of the input power. It is also proportional to the squared length of the nonlinear medium in the absence of wave mismatch (i.e. $\Delta k = 0$). It should to be noted that Eqns (75), (76) are valid only for low conversion efficiency ($P_o/P_p \leq\approx 1\%$).

The following parameters were used to estimate the 3HG efficiency in Si:P: $n_i = 3.41538$ at 4 THz and $n_o = 3.41534$ at 12 THz (RT), $\chi^{(3)} = 2.9\cdot 10^{-12}$ (V/m)^2 for a donor concentration $N = 10^{17}$ cm^{-3}. Using $L = 1.4$ cm, $d = 1$ mm and pump power of 1 W one can get the conversion efficiency of 1%.

VIII. EFFECT OF PHONON RELAXATION AND MULTIPHOTON IONIZATION

In this section we discuss processess that may possibly reduce the 3HG efficiency. Losses due to dephasing by phonon scattering may become important if the time spent in the intermediate states exceeds the phonon lifetime. Since the inverse of the former is given approximately by the detuning ($\Delta f \Delta t \geq 1/2\pi$) and the inverse phonon-limited width $1/\pi T_2 = 1$ GHz [11, 12], this loss is negligible for much of the spectrum.

Another nonlinear process that may lower the 3HG efficiency is multiphoton ionization [13] since it reduces the population of the donors in the ground state and creates additional decoherence. When $\omega = \bar{\omega}_{2p}/3$ for example, a four photon absorption takes the electron to the continuum. Here we estimate this ionization in Si:P using the implicit summation method.

The N-photon ionization rate can be estimated if the wavefunction of the ejected electron in the continuum is known [13]. We use the following approximation for the continuum wavefunction in Si:P: In the stretched frame given by the transformation of Eqn (9) we replace the asymmetric potential $1/r'\sqrt{1-(1-\gamma)(\cos\theta')^2}$ by its angular average κ/r' where

$$\kappa = \frac{1}{2}\int_0^{\pi} \frac{\sin\theta' d\theta'}{\sqrt{1-(1-\gamma)(\cos\theta')^2}} = \frac{\arctan\left(\sqrt{1/\gamma - 1}\right)}{\sqrt{1-\gamma}}, \tag{77}$$

so that the potential is spherical in the stretched frame. Assuming that the energy of the electron in the continuum is within a few tens meV from the conduction band minima, we can again employ effective mass theory. The envelope function of an ejected electron with wavenumber $\boldsymbol{k'}$ in the stretched frame in valley μ is given by $\Psi_\mu(\boldsymbol{r'}) = e^{i\boldsymbol{k'_\mu}\cdot\boldsymbol{r'}} \sum_{l=0}^{\infty} F_{\boldsymbol{k'},l,\mu}(\boldsymbol{r'})$ where

$$F_{\boldsymbol{k'},l,\mu}(\boldsymbol{r'}) = 4\pi e^{i\alpha_l} \sum_{m=-l}^{l} R_{k',l}(r') Y_l^m(\theta',\phi') Y_l^{m^*}(\Omega_{k'}), \tag{78}$$

where $k' = \sqrt{2m_t E}/\hbar = \sqrt{2E/E_H}/a_B$ with E the energy of the ejected electron, $\Omega_{k'}$ the solid angle of the ejected electron in the stretched frame, $\alpha_l = l\pi/2 + \arg[\Gamma(l+1-i\kappa/k'a_B)]$, and $R_{k',l}(r')$ is the radial wavefunction

$$R_{k',l}(r') = e^{\pi\kappa/2k'a_B} \left| \frac{\Gamma(l+1-\kappa/k'a_B)}{(2l+1)!} \right| (2k'r')^l e^{-ik'r'} F(i\kappa/k'a_B + l + 1, 2l+2, 2ik'r'), \tag{79}$$

where F is the confluent hypergeometric function of the first kind. These functions are taken from Ref. [13] and scaled to account for the factor κ.

The total transiton rate for multiphoton ionization in hydrogen is derived in Ref. [13]. Generalizing it for Si:P, taking into account the valley degeneracy, we obtain the total rate from the $1sA_1$ ground state to the continnuum manifold with energy E

$$w^{(N)} = \frac{1}{(2\pi)^2} \left(\frac{2\pi\alpha_{\rm fs}}{\sqrt{\epsilon_r}}\right)^N \left(\frac{I_m}{I_a}\right)^N \frac{E_H}{\hbar} |K^{(N)}|^2 (k'a_B), \tag{80}$$

where $K^{(N)}$ is a dimensionless matrix element given by

$$|K_{fg}^{(N)}|^2 = (1/a_B^3) \sum_{\mu=1}^{6} \int d\Omega_{k'} \left| \sum_{l=0}^{\infty} \langle F_{\boldsymbol{k'},l,\mu}| \zeta |F_{N-1,\mu}\rangle \right|^2 = \frac{(4\pi)^2}{a_B^3} \sum_{\mu=1}^{6} \sum_{l=0}^{\infty} \sum_{m=-l}^{l} \left| \sum_{\Delta m = 0,\pm 1} \langle f_{k',l,m}| \zeta_{\Delta m} |f_{N-1,m-\Delta m,\mu}\rangle \right|^2, \tag{81}$$

where $F_{N-1,\mu}$ are the envelope functions of the $(N-1)$th intermediate state defined in Eqn (24),$f_{N-1,m,\mu}$ its component in Eqn (29), $\zeta_{\Delta m}$ the component of the polarization operator defined in Eqn (52), and $|f_{k',l,m}\rangle$ are the 2D envelope functions

$$f_{k',l,m}(\theta',\phi') = R_{k',l}(r') \sqrt{\frac{(2l+1)}{4\pi}\frac{(l-m)!}{(l+m)!}} P_l^m(\cos\theta'), \tag{82}$$

where P_l^m are the associated Legendre polynomials.

For the 3ω transition to the average frequency of the $2p_0$ and $2p_\pm$ level in Si:P, a 4ω absorption takes the electron to the continuum with the energy $E \approx 0.237 E_H = 9.46$ meV. We find that, for a sample with $n_{\rm 3D} = 5\times 10^{15}$ cm^{-3},

the ionization rate is $w = 3.17\ \mathrm{s}^{-1}$ for $I_m = 10\ \mathrm{kW/cm}^2$. This simply means that the pulses must be kept significantly shorter than a second to avoid significant ionization.

[1] W. Kohn and J. M. Luttinger, Phys. Rev. **98**, 915 (1955).

[2] A. L. Saraiva, A. Baena, M. J. Calderón, and B. Koiller, J. Phys.: Condens. Matter **27**, 154208 (2015).

[3] R. A. Faulkner, Physical Review **184**, 713 (1969).

[4] B. Pajot, *Optical Absorption of Impurities and Defects in Semiconducting Crystals: Hydrogen-Like Centres* (Springer Berlin Heidelberg, 2010) p. 143, Table 5.9.

[5] K. Shindo and H. Nara, Journal of the Physical Society of Japan **40**, 1640 (1976).

[6] R. W. Boyd, *Nonlinear Optics*, Vol. 1 (Academic Press, 2008) p. 640, Eqns 3.2.23 and 3.2.37.

[7] P. D. Maker and R. W. Terhune, Phys. Rev. **148**, 990 (1966).

[8] Y. R. Shen, *The Principles of Nonlinear Optics*, 3rd ed. (Wiley-Interscience, 2002) p. 576.

[9] G. H. C. New and J. F. Ward, Phys. Rev. Lett. **19**, 556 (1967).

[10] R. Miles and S. Harris, IEEE J Q Electron. **9**, 470 (1973).

[11] M. Steger, A. Yang, D. Karaiskaj, M. L. W. Thewalt, E. E. Haller, J. W. Ager, M. Cardona, H. Riemann, N. V. Abrosimov, A. V. Gusev, A. D. Bulanov, A. K. Kaliteevskii, O. N. Godisov, P. Becker, and H.-J. Pohl, Phys. Rev. B **79**, 205210 (2009).

[12] P. T. Greenland, S. A. Lynch, A. F. G. van der Meer, B. N. Murdin, C. R. Pidgeon, B. Redlich, N. Q. Vinh, and G. Aeppli, Nature **465**, 1057 (2010).

[13] H. B. Bebb and A. Gold, Physical Review **143**, 1 (1966).
